# Blood miRNA levels associated with ADHD traits in children across six European birth cohorts

**DOI:** 10.1186/s12888-023-05199-5

**Published:** 2023-09-25

**Authors:** Lene B. Dypås, Nur Duale, Ann-Karin Olsen, Mariona Bustamante, Lea Maitre, Geòrgia Escaramis, Jordi Julvez, Sofia Aguilar-Lacasaña, Sandra Andrusaityte, Maribel Casas, Marina Vafeiadi, Regina Grazuleviciene, Barbara Heude, Johanna Lepeule, Jose Urquiza, John Wright, Tiffany C. Yang, Martine Vrijheid, Kristine B. Gützkow

**Affiliations:** 1 Division of Climate and Environmental Health, Norwegian Institute of Public Health, Oslo, Norway; 2 Institute for Global Health (ISGlobal), Barcelona, Spain; 3 Universitat Pompeu Fabra (UPF), Barcelona, Spain; 4 Centro de Investigación Biomédica en Red de Epidemiología y Salud Pública (CIBERESP), Madrid, Spain; 5 Department of Biomedical Sciences, Institute of Neuroscience, University of Barcelona, Barcelona, Spain; 6 Clinical and Epidemiological Neuroscience (NeuroÈpia), Institut d’investigació Sanitària Pere Virgili (IISPV), Reus, Catalonia Spain; 7 Department of Environmental Science, Vytautas Magnus University, Kaunas, Lithuania; 8 Department of Social Medicine, School of Medicine, University of Crete, Heraklion, Greece; 9 Centre of Research in Epidemiology and Statistics (CRESS), Inserm, Université de Paris, Paris, France; 10 Université Grenoble Alpes, INSERM, CNRS, Institute for Advanced Biosciences (IAB), Team of Environmental Epidemiology Applied to Development and Respiratory Health, La Tronche, France; 11 Bradford Teaching Hospitals NHS Foundation Trust, Bradford, UK

**Keywords:** ADHD, MicroRNA, Cohort study, Biomarkers

## Abstract

**Background:**

Attention-deficit/hyperactivity disorder (ADHD) is a prevalent and highly heritable neurodevelopmental disorder of major societal concern. Diagnosis can be challenging and there are large knowledge gaps regarding its etiology, though studies suggest an interplay of genetic and environmental factors involving epigenetic mechanisms. MicroRNAs (miRNAs) show promise as biomarkers of human pathology and novel therapies, and here we aimed to identify blood miRNAs associated with traits of ADHD as possible biomarker candidates and further explore their biological relevance.

**Methods:**

Our study population consisted of 1126 children (aged 5–12 years, 46% female) from the Human Early Life Exposome study, a study spanning six ongoing population-based European birth cohorts. Expression profiles of miRNAs in whole blood samples were quantified by microarray and tested for association with ADHD-related measures of behavior and neuropsychological functions from questionnaires (Conner’s Rating Scale and Child Behavior Checklist) and computer-based tests (the N-back task and Attention Network Test).

**Results:**

We identified 29 miRNAs significantly associated (false discovery rate < .05) with the Conner’s questionnaire-rated trait hyperactivity, 15 of which have been linked to ADHD in previous studies. Investigation into their biological relevance revealed involvement in several pathways related to neurodevelopment and function, as well as being linked with other neurodevelopmental or psychiatric disorders known to overlap with ADHD both in symptomology, genetic risk, and co-occurrence, such as autism spectrum disorder or schizophrenia. An additional three miRNAs were significantly associated with Conner’s-rated inattention. No associations were found with questionnaire-rated total ADHD index or with computer-based tests.

**Conclusions:**

The large overlap of our hyperactivity-associated miRNAs with previous studies on ADHD is intriguing and warrant further investigation. Though this study should be considered explorative and preliminary, these findings contribute towards identifying a set of miRNAs for use as blood-based biomarkers to aid in earlier and easier ADHD diagnosis.

**Supplementary Information:**

The online version contains supplementary material available at 10.1186/s12888-023-05199-5.

## Background

Attention-deficit/hyperactivity disorder (ADHD) is a childhood-onset neurodevelopmental disorder characterized by inattention and/or hyperactivity and impulsivity to a degree that interferes with normal functioning or development [1]. Prevalence in the worldwide population is estimated at 5.9% among youths and 2.5% among adults [2]. The disorder is associated with e.g., impaired social functioning, lower academic achievements, substance abuse and criminality, as well as an increased risk of several somatic illnesses, injury, and comorbid psychiatric disorders, leading to increased healthcare costs for patients and their family members [2]. Meta-analyses of twin studies on ADHD estimate a mean heritability of 74% [3], indicating substantial genetic involvement. Still, no single gene variant has been identified to confer considerable risk of ADHD, instead, several genes each contribute with a small increase in risk and ADHD has been noted as a polygenic and biologically complex disorder [4, 5]. Studies suggest an interplay of genetic and environmental factors, where epigenetic mechanisms have an important role [6, 7].

MicroRNAs (miRNA) are a class of small non-coding RNAs that regulate gene expression post-transcriptionally by pairing with target mRNAs to promote cleavage or translational repression [8]. They are involved in almost every biological processes and have been identified as key players in central nervous system development [9], prompting interest in exploring their role in the etiology of neurodevelopmental or psychiatric disorders, such as schizophrenia, autism spectrum disorder (ASD), bipolar disorder, and major depression disorder [10, 11]. Efforts to investigate their role in ADHD have mainly focused on biomarker identification and treatment response monitoring [12–14], while some studies have included mRNA expression levels, targeting of ADHD related genes identified from databases, grey matter volume, chronotype, and genetic variants, alongside their investigations of miRNAs [15–19]. A handful of studies report specific miRNAs associated with ADHD as promising biomarkers, but so far there is little overlap in results across studies, different analysis methods are used without standardization, and various sample types are utilized, such as whole blood, white blood cells, plasma, serum etc. Most studies also have relatively small study populations and only investigate a few select miRNAs [20]. Additionally, blood, or other accessible sample types, are needed as proxy for brain tissue in human studies on neurodevelopmental disorders. Though the blood–brain miRNA expression relationship is not yet well understood, the hypothesis is that peripheral blood is useful to reflect states in the brain, and a recent study on baboons found significant correlation of several miRNAs between brain regions and peripheral blood mononuclear cells [21]. Interestingly, the correlates were enriched in miRNAs expressed in immune cell types known as markers of neuroinflammation, and inflammation has long been suspected as part of ADHD pathogenesis [22].

ADHD presents with a heterogeneous phenotype across individuals, gender, and age [1, 23], and diagnosis can be challenging. This is especially true for early diagnosis, as it relies heavily on how the parents perceive their child and early presenting ADHD can be misinterpreted as part of the natural developmental process in preschool children [24]. Identifying a set of blood miRNAs specific to ADHD would therefore be of great value in several ways; as an efficient and minimally invasive diagnostic or prognostic biomarker for earlier diagnosis and better lifetime prognosis, to help elucidate etiology, and perhaps lead to personalized medication and other new therapeutic strategies [9, 25].

In this study we aimed to identify blood miRNAs associated with ADHD-related traits as possible biomarker candidates and further map the biological function of those miRNAs. Among challenges mentioned above were small study populations and inclusion of very few select miRNAs. One possibility to mitigate this is by utilizing large population-based cohorts, and for those cohorts to include miRNA analysis to their inventory. Here, we take the opportunity provided by the Human Early Life Exposome (HELIX) study [26], spanning six longitudinal population-based European birth cohorts, to investigate miRNA expression and ADHD-related traits in a large and geographically diverse population.

## Materials and methods

### Study population

The Human Early Life Exposome (HELIX) study is a collaboration across six established and ongoing longitudinal population-based European birth cohorts [26]: the Born in Bradford (BiB) study in the UK [27], the Étude des Déterminants pré et postnatals du développement et de la santé de l’Enfant (EDEN) study in France [28], the INfancia y Medio Ambiente (INMA) cohort in Spain [29], the Kaunas cohort (KANC) in Lithuania [30], the Norwegian Mother, Father and Child Cohort Study (MoBa) [31], and the RHEA Mother–Child Cohort study in Crete, Greece [32]. HELIX is and expansive cohort study focused on environmental exposures together with molecular profiles and neurodevelopment in a general population. Within HELIX, a subcohort of 1301 children was selected to participate in a follow-up examination between ages 5–12. These follow-up visits, conducted in 2014 and 2015 across all six study centers, included questionnaires, clinical examination, neurodevelopmental tests, and biological sample collection, with miRNA expression profile data available for 1126 children making up our study population. For more information on HELIX or the subcohort inclusion criteria, see Maitre et al. [26]. Further exclusion criteria were children with missing values for our chosen measures of ADHD-related traits (described below) and children using ADHD medication, as ADHD medication use is expected to target our chosen measures of behavior and neuropsychological functions and has been shown to affect expression levels of miRNAs differentially expressed between ADHD cases and controls [12]. Other medication use was not excluded, as we do not expect it to systematically target ADHD-related behavior or miRNAs specific for ADHD.

### Measures of behavior and neuropsychological functions

The HELIX subcohort data includes two computer-based tests and two parent-rated questionnaires with information regarding ADHD-related traits or symptoms: The N-back task, the Attention Network Test (ANT), Conner’s Rating Scale, and Child Behavior Checklist (CBCL). The N-back task is a computer-based test to assess working memory [33]. We chose the 3-back condition with numbers as stimuli and used the measure *d* prime (*d’*), which is the difference in hit rate and false alarm rate, allowing a distinction of signal and noise. A higher *d’* indicates better signal detection, i.e., better working memory. ANT measures the cognitive domain attention function [33]. Here we chose four measures; i) hit reaction time standard error (HRT-SE), which is response speed consistency throughout the test as a measure of inattentiveness, ii) incorrect responses (zeroes), as a measure of impulsivity, iii) omission errors (failure to respond), as a measure of selective attention, and iv) conflict score, as a measure of the executive attention network, which is involved in solving conflict between neural systems and in regulating thoughts and feelings. Optimal performance is reflected in low scores for all four outcomes. The 27-item short form of Conner’s Rating Scale [34] provided an ADHD index, along with two sub-scores categorized as hyperactivity and cognitive problems/inattention. We included these sub-scores in our analyses to reflect two of the main ADHD presentations, predominantly hyperactive and predominantly inattentive. The 99-item CBCL/6–18 version for children also provided a score for total ADHD problems [35]. Low scores indicate lighter problem load in both questionnaires. The questionnaires were completed by parents within a week of the follow-up visits for the subcohort.

### Blood miRNA expression

RNA was extracted from whole blood samples collected in Tempus Blood RNA Tubes [36] using the MagMAX for Stabilized Blood Tubes RNA Isolation kit (Thermo Fisher Scientific, USA). Extraction was done by order of arrival, i.e., by cohort. miRNA expression levels were quantified with the SurePrint Human miRNA Microarray rel. 21 (Agilent Technologies, USA) in two rounds of 1126 and 216 samples (1087 and 180 unique HELIX samples), at the Genomics Core Facility at the Centre for Genomic Regulation (CRG, Spain). Within each round, samples were randomized by sex and cohort. Expression levels were normalized using the least variant set method [37] with background correction by the Normexp method [38], followed by log_2_ transformation. miRNAs were annotated using Agilent annotation (“Annotation_70156”) combined with additional information from miRbase v21. Additional correction of blood cell composition and batch effect was done with the surrogate variable analysis (SVA) standard method [39]. SVA was performed for each neurodevelopmental measure separately, and we obtained the residuals of surrogate variables (SVs) while protecting sex, age, and cohort, with 23 SVs calculated for the N-back test measure, and 25 for all other neurodevelopmental measures. See Additional file 2, Fig. S1 for principal component analysis showing effect of SVA correction on laboratory processing round. After quality control and relevant processing steps the dataset consisted of 1436 autosomal miRNAs. We set a miRNA call rate of > 70%, resulting in 308 miRNAs for subsequent analysis. Detailed information is included in Additional file 1. This protocol was previously described by Vives-Usano et al. [40] for round 1 samples only.

### Statistical analyses

Our nine neurodevelopmental variables were age-adjusted through linear regression as a function of age and using the resulting residuals as replacement values. Data from Conner’s and CBCL were heavily right-skewed due to the nature of questionnaire data measuring deviations from the average population on only one end of the scale and with bounded response. ANT zeros and omissions, being count data with zero-inflation, had similar distributions. The residuals were thus transformed closer towards normality using Tukey’s Ladder of Power in the rcompanion R package and re-scaled with mean = 100 and a standard deviation (SD) equal to the residuals’ SD prior to transformation. Bivariate association analysis was performed between the variables age, sex, cohort, child’s ancestry, socioeconomic status (SES) (family affluence scale, FAS II [41], i.e., subjective wealth), maternal age and maternal education, and our nine neurodevelopmental measures. We used Spearman’s rank correlation for all variables except cohort, where one-way Analysis of Variance (ANOVA) was applied. Associations with age were investigated using the raw data, prior to transformation.

Linear regression models were applied to investigate the relationship between miRNA expression profiles and our selected neurodevelopmental variables using the limma R package [38], with sex and cohort included in the models as covariates. Note that the effect of age was already adjusted for. The Benjamini–Hochberg method for multiple testing correction was applied with a false discovery rate (FDR) < .05. We contrasted our findings with previous literature, based on a search in PubMed (see Additional file 1), both through direct comparison and enrichment analyses using the Chi-square or Fisher’s exact test. Sensitivity analyses were performed on the models by; i) analyses stratified by cohort, ii) analyses stratified by sex, iii) analyses stratified by or with laboratory processing round included as covariate, and iv) analyses with SES included as covariate. Missing values for SES were replaced by imputation using the R package missForest [42].

### Gene targets and KEGG pathways

Mienturnet (microRNA-target enrichment and network-based analysis) [43] with target validation from miRTarBase was used to predict genes targeted by miRNAs found significant from limma analyses. We further analyzed these miRNA-target interactions with the R package miRmapper to build a clustering dendrogram based on gene target similarity [44]. To gain further insight into the functional effects of the miRNAs identified in this study, we performed functional enrichment analysis with DIANA mirPath v.3 [45] using validated targets from TarBase v7. Analysis was run as genes union with FDR correction and significance set to *p* < 0.05.

## Results

### Demographic characteristics

The study population and its characteristics across cohorts, including scores for measures of behavior and neuropsychological functions, is presented in Table 1. There were slightly more male (54%) than female children, a trend consistent across cohorts with no significant difference (*p* = 0.9273) and EDEN being the most skewed towards male (57.5%). Median child age overall was 8.2 years but varied significantly (*p* < .0001) by cohort: BiB = 6.6, EDEN = 10.8, INMA = 9.0, KANC = 6.4, MoBa = 8.5, and RHEA = 6.5. With exception to the BiB cohort, which had 55.4% children of non-European ancestry from mainly Pakistani origins, there was little variation in the self-reported ancestry, as 87.9% of the study population were of European ancestry. Mean maternal age was relatively similar across cohorts, though still a statistically significant difference (*p* < .0001). MoBa stood out as the cohort with the highest level of maternal education, with 76.7% highly educated vs. 46.7% in the total population, and together with EDEN they also had the highest SES rating.

**Table 1:** Study population characteristics and neuropsychological function scores for the HELIX subcohort

	**BiB**	**EDEN**	**INMA**	**KANC**	**MoBa**	**RHEA**	**Total**	***P***
	**(*N* = 166)**	**(*N* = 87)**	**(*N* = 314)**	**(*N* = 153)**	**(*N* = 249)**	**(*N* = 157)**	**(*N* = 1126)**	
**Child sex**								
Male	91 (54.8%)	50 (57.5%)	164 (52.2%)	84 (54.9%)	131 (52.6%)	88 (56.1%)	608 (54.0%)	0.9273^a^
Female	75 (45.2%)	37 (42.5%)	150 (47.8%)	69 (45.1%)	118 (47.4%)	69 (43.9%)	518 (46.0%)	
**Child age**								
Years	6.64 [6.15, 7.33]	10.8 [9.28, 12.0]	8.99 [7.56, 10.7]	6.41 [5.44, 7.81]	8.45 [6.92, 9.80]	6.46 [6.02, 7.54]	8.17 [5.44, 12.0]	< .0001^b^
**Child ancestry**								
European	74 (44.6%)	87 (100%)	281 (89.5%)	153 (100%)	238 (95.6%)	157 (100%)	990 (87.9%)	< .0001^a^
Non-European	92 (55.4%)	0 (0%)	33 (10.5%)	0 (0%)	11 (4.4%)	0 (0%)	136 (12.1%)	
**SES**								
Low	47 (28.3%)	0 (0%)	30 (9.6%)	20 (13.1%)	3 (1.2%)	27 (17.2%)	127 (11.3%)	< .0001^a^
Medium	73 (44.0%)	18 (20.7%)	115 (36.6%)	84 (54.9%)	69 (27.7%)	76 (48.4%)	435 (38.6%)	
High	46 (27.7%)	69 (79.3%)	166 (52.9%)	48 (31.4%)	177 (71.1%)	54 (34.4%)	560 (49.7%)	
(Missing)	0 (0%)	0 (0%)	3 (1.0%)	1 (0.7%)	0 (0%)	0 (0%)	4 (0.4%)	
**Maternal age**								
Years	28.0 [16.0, 42.0]	30.1 [20.2, 40.8]	32.1 [18.2, 43.0]	28.5 [19.2, 43.5]	32.0 [24.0, 43.0]	30.9 [17.0, 42.3]	31.0 [16.0, 43.5]	< .0001^b^
**Maternal education**								
Low	76 (45.8%)	8 (9.2%)	75 (23.9%)	9 (5.9%)	0 (0%)	7 (4.5%)	175 (15.5%)	< .0001^a^
Medium	21 (12.7%)	33 (37.9%)	117 (37.3%)	56 (36.6%)	48 (19.3%)	90 (57.3%)	365 (32.4%)	
High	51 (30.7%)	44 (50.6%)	97 (30.9%)	85 (55.6%)	191 (76.7%)	58 (36.9%)	526 (46.7%)	
(Missing)	18 (10.8%)	2 (2.3%)	25 (8.0%)	3 (2.0%)	10 (4.0%)	2 (1.3%)	60 (5.3%)	
**Conner’s**								
ADHD Index	98.2 [66.4, 134]	99.5 [82.1, 135]	104 [74.9, 136]	107 [65.2, 130]	91.8 [70.8, 132]	104 [67.5, 136]	101 [65.2, 136]	< .0001^b^
Hyperactivity	97.8 [65.9, 134]	102 [92.3, 127]	101 [84.8, 134]	107 [54.5, 135]	89.3 [74.5, 122]	102 [63.7, 130]	98.7 [54.5, 135]	< .0001^b^
Inattention	91.6 [79.4, 135]	93.6 [82.4, 133]	105 [80.7, 134]	111 [78.7, 131]	92.3 [80.1, 134]	98.4 [79.4, 134]	99.3 [78.7, 135]	< .0001^b^
(Missing)	0 (0%)	0 (0%)	3 (1.0%)	0 (0%)	0 (0%)	4 (2.5%)	7 (0.6%)	
**CBCL**								
ADHD Problems	102 [73.0, 126]	101 [82.1, 136]	105 [77.6, 135]	107 [70.5, 130]	90.2 [75.6, 129]	102 [73.5, 134]	101 [70.5, 136]	< .0001^b^
(Missing)	0 (0%)	0 (0%)	2 (0.6%)	1 (0.7%)	0 (0%)	2 (1.3%)	5 (0.4%)	
**ANT**								
HRT-SE	104 [66.1, 126]	106 [79.7, 141]	99.2 [64.5, 142]	105 [68.7, 132]	95.0 [63.6, 136]	99.8 [61.5, 129]	100 [61.5, 142]	< .0001^b^
Zeros	96.6 [58.2, 143]	106 [98.2, 139]	100 [82.8, 128]	91.5 [28.7, 148]	99.6 [78.0, 141]	97.5 [57.3, 147]	100 [28.7, 148]	< .0001^b^
Omissions	96.8 [59.6, 145]	111 [102,126]	102 [84.1, 145]	103 [40.8, 167]	96.4 [73.6, 132]	91.3 [45.3, 150]	100 [40.8, 167]	< .0001^b^
Conflict	99.3 [51.0, 172]	100 [45.1, 134]	99.2 [58.8, 149]	99.8 [27.0, 136]	98.9 [69.8, 204]	97.9 [-5.19, 172]	99.3 [-5.19, 204]	0.7730^b^
(Missing)	3 (1.8%)	6 (6.9%)	3 (1.0%)	0 (0%)	0 (0%)	0 (0%)	12 (1.1%)	
**N-back**								
*d’*	98.3 [68.5, 147]	95.9 [62.8, 131]	101 [60.8, 142]	NA	102 [63.6, 139]	102 [68.4, 149]	101 [60.8, 149]	0.0864^b^
(Missing)	26 (15.7%)	7 (8.0%)	8 (2.5%)	153 (100%)	10 (4.0%)	15 (9.6%)	219 (19.4%)	

Presented are N (%) or median [min, max]. Measures of behavior and neuropsychological function are presented after transformation of raw data

*BiB* Born in Bradford (UK), *EDEN* Étude des Déterminants pré et postnatals du développement et de la santé de l’Enfant (France), *INMA* INfancia y Medio Ambiente (Spain), *KANC* Kaunas cohort (Lithuania), *MoBa* Norwegian Mother, Father and Child Cohort Study, *RHEA* Mother–Child Cohort study in Crete (Greece), *SES* Socioeconomic status, *CBCL* Child Behavior Checklist, *ANT* Attention Network Test, *HRT-SE* Hit reaction time standard error

*P*-values for significant difference between cohorts

^a^*p*-values from chi-square test of homogeneity

^b^*p*-values from one-way ANOVA

Fourteen subjects were removed due to ADHD medication use: one from EDEN and 13 from INMA. Of these 14 subjects, 11 were male, and the median age for all 14 was 9.3 years with a range of 8.1–11.2. For the N-back task there was a total of 219 subjects with missing values, including all participants in KANC due to technical issues, leaving a study population of 894 subjects. The rest: ANT, Conner’s, and CBCL, together had a total of 23 subjects with missing values, and thus a study population of 1089 subjects.

### Bivariate association analysis

Prior to transformation, all measures of behavior and neuropsychological functions, except for Conner’s inattention, correlated with age at a significant level (*p* < 0.0001). This relationship was moderate in strength (correlation coefficient, *r*_*s*_ = .40-.59) for ANT HRT-SE, zeros, and omissions; weak (*r*_*s*_ = .20-.39) for ANT conflict, N-back, and Conner’s hyperactivity; and very weak (*r*_*s*_ < .20) for Conner’s ADHD index and CBCL ADHD problems (Additional file 2, Fig. S2). After age-adjusting and Tukey transformation, the variable cohort correlated most with our measures of behavior and neuropsychological functions (Table 2). Only ANT conflict and N-back were not significantly correlated with cohort. Sex, SES, maternal age, and maternal education were all significantly correlated with only half or less of our measures of behavior and neuropsychological functions, child’s ancestry with none, and the strength of these relationships were all very weak. Notably, the strongest relationships with sex were observed for the three measures from Conner’s, and in questionnaire data, female subjects were consistently rated with fewer ADHD-related problems compared to male subjects (Additional file 2, Fig. S3).

**Table 2:** Bivariate association between measures of behavior and neuropsychological functions and selected variables describing study population characteristics

**Variable**	**ANT**					**Conner’s Rating Scale**			**CBCL**
	**HRT-SE**	**Zeros**	**Omissions**	**Conflict**	**N-back**	**ADHD index**	**Inattention**	**Hyperactivity**	**ADHD problems**
**Cohort**^a^									
BiB	101.9 (14.1)***	98.9 (20.2)***	97.6 (19.4)***	100.9 (18.2)	97.9 (15.4)	96.1 (17.1)***	96.0 (15.4)***	95.9 (18.4)***	97.8 (16.1)***
EDEN	107.4 (15.6)	107.5 (6.5)	111.9 (3.9)	101.2 (9.4)	97.1 (14.5)	103.9 (11.9)	99.1 (13.0)	106.6 (9.7)	103.7 (11.8)
INMA	99.7 (15.1)	100.5 (8.2)	102.3 (6.4)	99.1 (10.2)	100.5 (15.6)	102.2 (15.1)	104.5 (14.8)	103.9 (13.0)	104.0 (14.1)
KANC	104.1 (12.4)	94.4 (20.7)	104.5 (21.4)	98.8 (16.2)	NA (NA)	104.3 (14.4)	107.9 (13.0)	105.2 (17.1)	104.5 (15.8)
MoBa	94.8 (15.1)	101.4 (10.9)	97.2 (7.5)	100.8 (13.8)	101.2 (13.9)	93.9 (13.9)	94.7 (13.3)	92.8 (8.7)	91.9 (12.8)
RHEA	99.1 (14.8)	99.5 (18.5)	91.9 (19.8)	100.1 (20.9)	100.6 (15.3)	103.5 (11.8)	96.9 (14.8)	100.1 (16.8)	101.6 (14.1)
**Child sex**	0.06	-0.12**	0.06	-0.11**	0.01	-0.15***	-0.15***	-0.14***	-0.07
**Child ancestry**	0.07	0.05	0.04	0.06	-0.03	-0.02	-0.04	-0.02	0.02
**SES**	-0.06	-0.01	0.00	-0.01	0.04	-0.11**	-0.05	-0.07	-0.12**
**Maternal age**	-0.10**	0.01	-0.06	-0.04	0.07	-0.10*	-0.05	-0.10*	-0.13***
**Maternal education**	-0.14***	-0.05	-0.10*	0.00	0.10*	-0.10*	-0.06	-0.14***	-0.20***

*BiB* Born in Bradford (UK), *EDEN* Étude des Déterminants pré et postnatals du développement et de la santé de l’Enfant (France), *INMA* INfancia y Medio Ambiente (Spain), *KANC* Kaunas cohort (Lithuania), *MoBa* Norwegian Mother, Father and Child Cohort Study, *RHEA* Mother–Child Cohort study in Crete (Greece), *ANT* Attention Network Test, *HRT-SE* Hit reaction time standard error, *N-back* The N-back task with 3-back numbers, *CBCL* Child Behavior Checklist, *SES* Socioeconomic status, *SD* Standard deviation

^a^ For cohort, *p*-values from one-way ANOVA; mean (*SD*) are shown

*p*-values based on Spearman rank coefficient; correlation coefficient is shown. * *p* < .01. ** *p* < .001. *** *p* < .0001

### miRNA expression profiles and neuropsychological functions

Linear regression models in limma identified statistically significant associations with the Conner’s sub-scores for hyperactivity and inattention, while no significant results were achieved for its ADHD index, CBCL, or measures from ANT and N-Back. Conner’s hyperactivity score was significantly associated with expression levels of 29 miRNAs (Table 3). All 29 miRNAs had lower expression levels for higher hyperactivity scores and effect sizes were small, with a log_2_ fold-change (defined here as the change in expression per unit of increase in the predictor variable) in the range -0.011 to -0.036. Three miRNAs were significantly differentially expressed for Conner’s inattention score: miR-4257 had lower expression levels for higher inattention scores, while miR-4443 and miR-3180-3p had higher expression levels (Table 3). Also here, effect sizes were small, with a log_2_ fold-change of -0.016, 0.020, and 0.020, respectively. Note that interpretation of effect sizes needs to account for the data having been transformed and re-scaled. Effect sizes will also appear smaller due to our measures of behavior and neuropsychological functions being on a continuous scale, as compared to a case–control study. Sensitivity analysis resulted in changes to significance, mainly loss of significance, as detailed in Additional file 3, Table S1. miRNAs retained as significant were the same as the 32 identified in our original models, with only a few exceptions.

**Table 3:** MiRNAs statistically significant for Conner’s hyperactivity or inattention score

	**miRNA**	**log**_**2**_**FC**	**95% CI**	**Adj. *p* value**
**Hyperactivity**	hsa-miR-144-3p	-0.0361	± 0.0220	0.0240
	hsa-miR-19a-3p^a^	-0.0278	± 0.0155	0.0148
	hsa-miR-142-3p^a^	-0.0268	± 0.0152	0.0154
	hsa-miR-101-3p^a^	-0.0252	± 0.0130	0.0148
	hsa-miR-29c-3p	-0.0251	± 0.0139	0.0148
	hsa-miR-29b-3p	-0.0244	± 0.0133	0.0148
	hsa-miR-29a-3p	-0.0222	± 0.0135	0.0240
	hsa-miR-19b-3p^a^	-0.0211	± 0.0133	0.0299
	hsa-miR-148b-3p^a^	-0.0192	± 0.0107	0.0148
	hsa-miR-30e-5p^a^	-0.0190	± 0.0105	0.0148
	hsa-miR-130a-3p^a^	-0.0185	± 0.0110	0.0224
	hsa-miR-150-5p^a^	-0.0182	± 0.0123	0.0423
	hsa-let-7i-5p^b^	-0.0175	± 0.0096	0.0148
	hsa-miR-181b-5p	-0.0168	± 0.0094	0.0148
	hsa-miR-186-5p	-0.0164	± 0.0091	0.0148
	hsa-miR-146b-5p	-0.0161	± 0.0102	0.0299
	hsa-miR-106b-5p^a^	-0.0157	± 0.0101	0.0300
	hsa-miR-942-5p^a^	-0.0156	± 0.0076	0.0148
	hsa-miR-107^a^	-0.0149	± 0.0085	0.0161
	hsa-miR-27a-3p^a^	-0.0144	± 0.0093	0.0300
	hsa-miR-140-3p^a^	-0.0136	± 0.0081	0.0225
	hsa-miR-181a-5p	-0.0135	± 0.0087	0.0300
	hsa-miR-24-3p^a^	-0.0127	± 0.0083	0.0322
	hsa-miR-92a-3p^a^	-0.0127	± 0.0076	0.0225
	hsa-let-7c-5p^b^	-0.0127	± 0.0081	0.0300
	hsa-miR-103a-3p^b^	-0.0123	± 0.0084	0.0461
	hsa-miR-342-5p	-0.0121	± 0.0083	0.0458
	hsa-miR-324-5p	-0.0119	± 0.0075	0.0299
	hsa-miR-378i	-0.0112	± 0.0074	0.0354
**Inattention**	hsa-miR-4257	-0.0159	± 0.0080	0.0282
	hsa-miR-4443	0.0197	± 0.0103	0.0282
	hsa-miR-3180-3p	0.0198	± 0.0107	0.0287

Significance at FDR < .05. Table sorted by log_2_FC. log_2_FC, log_2_ fold-change defined as change in expression per unit of increase in the predictor variable; *95% CI* 95% confidence interval for log_2_FC, *Adj. p value P* value adjusted by FDR correction

^a^ miRNAs associated with ADHD in previous literature

^b^ miRNAs belonging to miRNA-families associated with ADHD in previous literature

A literature search in PubMed identified 56 miRNAs whose expression levels have been previously linked with ADHD using various sample matrices [15, 19, 46–49] (Additional file 3, Table S2), and 36 of these were present in our 308-miRNA expression matrix. Our set of 29 miRNAs differentially expressed for hyperactivity included 15 of those from previous literature (Table 3). Additionally, three others belong to families of miRNAs associated with ADHD. From direct comparison alone this is more overlap than we expected from random chance. Chi-square (*p* = 5.9e-14) and Fisher’s exact tests (*p* = 9.6e-10) confirmed this, and the expected overlap would be to find three of our 29 significant miRNAs in the literature list.

**Fig. 1:**
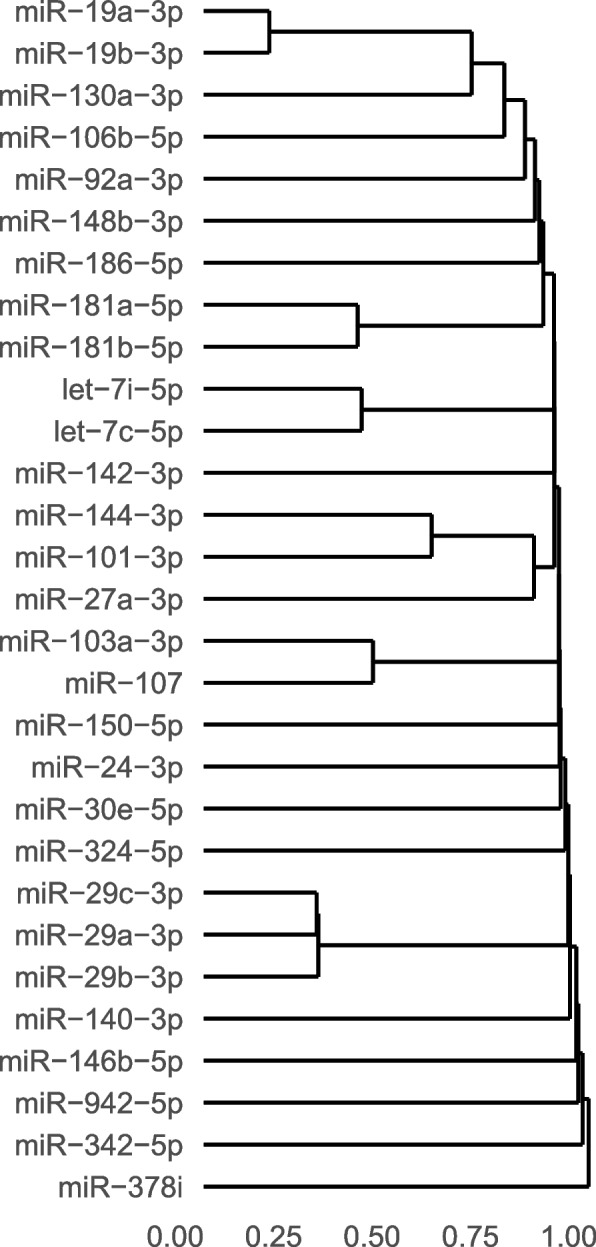
Clustering dendrogram of differentially expressed miRNAs significantly associated with the hyperactivity score. Based on the similarity of the miRNAs’ Jaccard index values to each other. Analyzed by the R-package miRmapper, see Da Silveira et al. (2018) [44] for calculations

In the clustering dendrogram based on gene target similarity for the 29 miRNAs identified for hyperactivity, miRNA-families clustered together (Fig. 1). These families are based on ancestors in the phylogenetic tree, suggesting a common sequence or structure configuration, and thus also similar physiological functions. The easily identifiable family clusters in the dendrogram are members of the let-7, miR-19, miR-181, and miR-29 families. Additionally, there were two members of the mir-15/107 family: miR-103a-3p, and miR-107.

Functional enrichment analysis of the genes targeted by our 29 miRNAs identified for hyperactivity revealed involvement in several KEGG pathways [50] relevant to neurodevelopment and function, such as axon guidance, neurotrophin signaling, and sphingolipid signaling; fatty acid metabolism, degradation, and biosynthesis; signaling pathways for the hormones insulin, prolactin, estrogen, and thyroid hormone; steroid biosynthesis, and glycosaminoglycan biosynthesis for keratan sulfate (Additional file 3, Table S3). We also performed functional enrichment analysis for genes targeted by the 15 miRNAs previously implicated in ADHD. In addition to the pathways mentioned above, the highly relevant pathway circadian rhythm was significant for this smaller set of gene targets, along with the vitamin B6 metabolism pathway (Additional file 3, Table S4).

## Discussion

In this study we have identified several miRNAs associated with hyperactivity, that have also been linked to ADHD in previous studies, as possible biomarker candidates. This large overlap, without pre-selection of miRNAs from literature, is an important contribution to the field. In addition, our study population was geographically diverse, spanning six European countries, with a relatively wide age-span. The results also highlight some of the challenges in the search for biomarkers of ADHD, surrounding the different presentations of the disorder, age of noticeable symptoms, and gender differences combined with societal expectations and masking.

Hyperactivity measured by the parent-rated questionnaire Conner’s Rating Scale was found associated with 29 miRNAs, 15 of which had already been linked to ADHD by other studies on blood-based samples or target prediction. In alignment with our findings, miR-106b-5p, miR-107, miR-24-3p, and miR-148b-3p have all been identified as downregulated in whole blood of ADHD cases compared to controls in previous studies [15, 51]. Another study on plasma samples identified miR-142-3p as downregulated in ADHD cases with psychiatric disorder history in their immediate family [17]. Recently, in a study based on literature and publicly available databases, we identified a set of ADHD-related genes along with 20 miRNAs as potential regulators of those genes, including miR-19a-3p, miR-19b-3p, and miR-24-3p [19]. This replication of results is especially exciting as our previous study was based on target-prediction, not analysis of biological samples from subjects. Analysis of publicly available data of expression in brain tissue for this ADHD-gene and -miRNA set also revealed that their expression patterns were dependent on developmental stage [19].

Further, we identified three miRNAs associated with the inattention trait of Conner’s Rating Scale, while no miRNAs were significantly differentially expressed for the total ADHD index. This highlights the importance of including ADHD presentation, as more and more research now explores the heterogeneity of ADHD, its varying presentations, or whether adult-persistent ADHD could be a separate subtype. The differences between the distinct presentations of ADHD have shown to contribute to poor separation of ADHD cases from neurotypical controls in performance on neuropsychological tests, and separation was greater when splitting cases into groups depending on type of presentation [52]. Findings of genetic associations with different presentations of ADHD further strengthens the hypothesis that there is a biological difference, and again, highlights the need to include this information in studies [53, 54].

The concordance of the hyperactivity trait with previous findings on miRNAs in ADHD, and not the inattentive trait, could in part be due to the higher likelihood of individuals with the combined or hyperactive presentation, with evident hyperactivity, to be referred for clinical evaluation, and thus having higher prevalence in clinical research [55]. More importantly, symptoms of hyperactivity and impulsivity can typically be observed as early as four years of age, and peak in severity at 7–8 years, while symptoms of inattention in general only becomes noticeable later, around 8–9 years of age [56, 57]. Symptoms of inattention could also go unnoticed by parents until demands put on the child increase and cause them to fail at school or in social interactions, as compared to high levels of hyperactivity and impulsivity that are more immediately troubling to caretakers at home. This delay in noticeable symptom impact is even more predominant in children with high IQ-scores or living in a highly structured environment [58]. As the median age of this study was eight years, and with relatively high SES-rating, one explanation could be that the parent-rated hyperactivity measure is more relevant and accurate for this population when compared to the inattention measure.

Limitations in the use of parent-rated questionnaires could also be relevant to the results seen in sensitivity analysis stratified by sex. There were no significant associations in the female population, while the male population retained two and four significant miRNAs for inattention and hyperactivity, respectively. Prosocial and compensatory behavior to mask impairments is to a greater extent seen in girls and women, and combined with gender biases due to stereotypical expectations, this could lead to underreporting or inconsistencies from parental observation of girls [59, 60]. This trend is observed in our questionnaire data, where female subjects were consistently rated with fewer ADHD-related problems compared to male subjects (Additional file 2, Fig. S3A-D), however we cannot conclude whether this is due to real differences between the sexes in this population or from bias in parental observation.

Efforts to identify a miRNA-based biomarker of ADHD have not yet produced robust findings validated by several studies, and most studies have used small populations, such as those already mentioned, with *N* = 29–104 [15, 17, 51]. ADHD and its traits, as well as other neurodevelopmental and psychiatric disorders, are complex and polygenic, so it can be hypothesized that a set of several miRNAs contributes to, or are an effect of, the overall etiology through small changes in each one, as opposed to monogenic conditions where there is great contribution and effect from one single source. Our findings and the large overlap with previous work are in line with this hypothesis and the feasibility of then building a multiple-miRNA model. One attempt at such a prediction model for ADHD was based on 13 miRNAs measured in white blood cell samples. They used a small study population as a training set (*N* = 122, AUROC (Area Under the Receiver Operating Characteristics): 0.94) and showed satisfactory results in classifying subjects in a testing set (*N* = 40, AUROC: 0.91) [61]. Six of our miRNAs were also included in this model. Later they replicated their results with 12 of the original 13 miRNAs, where all were differentially expressed at a significant level between ADHD cases and controls (*N* = 228) and their model attained an AUROC of 0.97, but without a testing set [48]. Wang et al. also raised concerns regarding their studies not accounting for different ADHD presentations and the limitation in their study population being selected from a small geographical area with no ethnic diversity.

This latest work by Wang et al. also included a follow-up after 12 months of methylphenidate treatment that identified miR-140-3p and miR-27a-3p as potential biomarkers of remission state during MPH treatment. Further, in a previous study they found gray matter volume to be negatively correlated with expression levels of miR-140-3p and miR-30e-5p [16], and followed this up now by observing that miR-140-3p facilitated growth of the HCN-2 human neuronal cell line by repressing apoptosis [48].

Several of our 29 hyperactivity-associated miRNAs belong to miRNA-families relevant for neurodevelopment and/or brain function. The ten members of the highly conserved miR-15/107 family includes miR-103a, and miR-107, and have, among other things, been implicated in cell division, stress response, and neurodegenerative disease [62]. Members of the miR-17 family, with miR-106b, have been identified as regulators of early development and stem cell differentiation [63]. Among some of the first discovered and most highly expressed miRNAs, are the let-7 family members, including let-7c and let-7i. Though most of our knowledge of their function is related to cancers, they also regulate important functions in the developing brain [64]. Let-7c regulates synaptic and neuronal function and is associated with major depression disorder [64, 65]. This family has also been associated with ADHD, as let-7d and let-7g have been implicated in several studies [12, 13, 61, 66]. Another interesting family of four is the miR-181 family, with miR-181a and miR-181b, implicated in embryo and CNS development and several neurodegenerative diseases [67]. Our miRNA-set also includes the 3p mature form of all three members of the miR-29 family, which are associated with neurodegenerative disease, as responders to environmental stress factors, mediators of the antidepressant effect of ketamine, and as essential regulators of brain maturation [68, 69].

miRNAs are also grouped by genome location, as physically adjacent clusters transcribed together, but not necessarily with similar targets. One of the most well-known miRNA clusters is the miR-17/92 cluster. The main miR-17/92 cluster includes miR-19a, miR-19b, and miR-92a from our hyperactivity-associated miRNA-set, while its paralogue cluster, miR-106b/25, also includes miR-106b. A second paralogue cluster, miR-106a/363, holds second loci for miR-19b and miR-92a. The miR-17/92 cluster is highly expressed in embryonic cells and is essential for normal development [70]. It has been implicated in immunity, neurodegenerative diseases, neuronal plasticity, as well as axonal outgrowth and guidance through modulation of PTEN protein levels [71]. Notably, these four miRNAs, plus miR-130a, are located together at the top of our clustering dendrogram, and they have all been previously linked with ADHD. Functional enrichment analysis also revealed that all five miRNAs target genes in almost all the pathways mentioned earlier as relevant to neurodevelopment and ADHD, including circadian rhythm and axon guidance. This group is of particular interest for further validation of the results in this study.

Neurodevelopmental and psychiatric disorders are known to overlap, both in symptomology, genetic risks, and co-occurrence [72, 73]. ASD, schizophrenia, bipolar disorder, and major depression disorder are all examples of diagnoses showing such overlap with ADHD, and almost all our 32 miRNAs are associated with one or more of these disorders (see Additional file 3, Table S5). ASD is especially well known for co-occurring with ADHD, and it has been reported that 1 in 8 youths with ADHD had co-occurring ASD [74]. Conversely, in ASD, ADHD is one of the most common comorbid disorders, with comorbidity rates around 60% [75]. The high number of previous associations to these related disorders further indicates that these miRNAs could have prominent roles in the etiology of ADHD, while the challenge then becomes finding an ADHD-specific signature.

The results of this study should be interpreted while considering its limitations. First, comparing or transferring our findings to studies on clinically assessed ADHD could be muddled by our lack of diagnosis. Here we separate and look at individual traits on a continuous scale in a general, healthy population, and these miRNAs may not be of the same importance for the full disorder presentation. In addition, while the separated Conner’s scores for hyperactivity and inattention contain several facets of these traits and to some extent represent main drivers in two of the three defined presentations of ADHD, the lack of full disorder evaluation may also be contributing to the small effect sizes. Secondly, due to sample size limitations we did not achieve robust findings, and in sensitivity analyses stratified by cohort or sex, the already relatively small population is split into even smaller groups. The combination of geographical diversity, age differences between cohorts, the expectation of our neurodevelopmental measures being associated with small changes in miRNA levels, and the temporally dynamic nature of miRNA expression driven by both internal and external environment, creates a need for larger study populations. In addition, it is warranted to consider the lack of consistency across cohorts. Despite great efforts to harmonize protocols across cohorts, there will always be some effects from sample and data collection at different labs, cultural differences, meals prior to testing etc. The study population characteristics, such as age, ancestry, SES, and maternal education were also inconsistent across cohorts, as previously mentioned (Table 1). Additionally, ratings for ADHD symptoms varied between cohorts. Comparing Conner’s scores between INMA and MoBa, two cohorts with similar median age, the median scores in MoBa are below the HELIX subcohort median, while INMA scores are above (Table 1). This, together with small sample size, may be contributing to what is observed in sensitivity analyses, with cohorts possibly driving results differently (Additional file 3, Table S1). For instance, when INMA is removed from the model all significance is lost, and it is the only cohort with significance remaining when analyzed separately. INMA is the largest of the six cohorts and it’s Conner’s scores are higher compared with the second largest cohort, MoBa. The results in this study should thus be considered exploratory and preliminary.

With these limitations taken into consideration, the lack of significant findings for the computer-based test measures could be related to the three main points listed above; lack of diagnosis, sample size limitations, and lack of consistency across cohorts. From the computer-based tests we isolate and evaluate one single continuous measure of a trait in a general healthy population. The changes in miRNA levels are thus expected to be very small, an effect further compounded by peripheral blood samples as a surrogate for brain tissue. In comparison, the questionnaires combine several measures, i.e., questions, to describe a somewhat broader behavior with limitations in many facets at the same time, possibly making it a stronger candidate for analysis in this context.

In conclusion, here we identified 32 miRNAs significantly associated with the ADHD-related traits hyperactivity (29 miRNAs) or inattention (3 miRNAs), but most importantly, 15 of these had already been linked to ADHD, an exciting overlap across studies. This study also highlights the usefulness of including information on distinct ADHD presentation. Further investigation of expression levels for these miRNAs in a case–control study, preferably with information about ADHD presentation, to validate our findings using quantitative PCR, is warranted. Future work should aim to identify a set of miRNAs to be used as biomarkers separating subjects with ADHD from neurotypicals, and such a signature will need to be robust against other co-occurring neurodevelopmental or psychiatric disorders. We are currently working on a case–control study investigating expression levels of selected miRNAs in cord blood plasma from children who have later been diagnosed with ADHD, along with samples collected from mothers and fathers during pregnancy. A selection of our 32 miRNAs will now be included. Investigating expression levels of these miRNAs at birth, by using cord blood, is important in the search for persistent biomarkers and to identify developmental processes regulated by these miRNAs during early development.

### Supplementary Information

**Additional file 1.** Additional details of methods.

**Additional file 2: Fig. S1.** PCA (principal component analysis) before and after SVA (surrogate variable analysis). **Fig. S2.** Correlation matrix for bivariate association between measures of behavior and neuropsychological functions and child’s age. **Fig. S3.** Behavioral and neuropsychological scores by sex.

**Additional file 3: Table S1.** Change in number of miRNAs significantly differentially expressed after sensitivity analyses. **Table S2.** miRNAs linked to ADHD in previous literature. **Table S3.** Functional enrichment analysis of 29 miRNAs significantly associated with hyperactivity. **Table S4.** Functional enrichment analysis of 15 miRNAs associated with hyperactivity/ADHD in this and previous studies. **Table S5.** MiRNAs statistically significant for Conner’s hyperactivity or inattention score, and their previous associations with neurodevelopmental or psychiatric phenotypes.

## Data Availability

The data that support the findings of this study are available from the HELIX project, but restrictions apply to the availability of these data, which were used under license for the current study, and so are not publicly available. Data are however available from the authors upon reasonable request and with permission from the HELIX project (https://projecthelix.eu).

## Abbreviations

ADHD: Attention-deficit/hyperactivity disorder
ANOVA: Analysis of variance
ANT: Attention Network Test
ASD: Autism spectrum disorder
AUROC: Area under the receiver operating characteristics
BD: Bipolar disorder
BiB: Born in Bradford
CBCL: Child Behavior Checklist
CSRS: Conner’s Rating Scale
EDEN: Étude des Déterminants pré et postnatals du développement et de la santé de l’Enfant
FDR: False discovery rate
HELIX: The Human Early Life Exposome project
HRT-SE: Hit reaction time standard error
INMA: INfancia y Medio Ambiente
KANC: The Kaunas cohort
log_2_FC: Log_2_ fold change
MDD: Major depression disorder
miRNA: MicroRNA
MoBa: The Norwegian Mother, Father, and Child Cohort Study
RHEA: The RHEA Mother–Child cohort study
SD: Standard deviation
SES: Socioeconomic status
SVA: Surrogate Variable Analysis
SVs: Surrogate variables
SZ: Schizophrenia

## References

[1] Faraone SV, Asherson P, Banaschewski T, Biederman J, Buitelaar JK, Ramos-Quiroga JA (2015). Attention-deficit/hyperactivity disorder. Nat Rev Dis Primers.

[2] Faraone SV, Banaschewski T, Coghill D, Zheng Y, Biederman J, Bellgrove MA, et al. The world federation of ADHD international consensus statement: 208 evidence-based conclusions about the disorder. Neurosci Biobehav Rev. 2021;128:789–818. 10.1016/j.neubiorev.2021.01.022 PMC8328933 33549739

[3] Faraone SV, Larsson H (2019). Genetics of attention deficit hyperactivity disorder. Mol Psychiatry.

[4] Demontis D, Walters RK, Martin J, Mattheisen M, Als TD, Agerbo E, et al. Discovery of the first genome-wide significant risk loci for attention deficit/hyperactivity disorder. Nat Genet. 2018;51:63–75. 10.1038/s41588-018-0269-7 PMC6481311 30478444

[5] Demontis D, Walters GB, Athanasiadis G, Walters R, Therrien K, Nielsen TT (2023). Genome-wide analyses of ADHD identify 27 risk loci, refine the genetic architecture and implicate several cognitive domains. Nat Genet.

[6] Kim JH, Kim JY, Lee J, Jeong GH, Lee E, Lee S (2020). Environmental risk factors, protective factors, and peripheral biomarkers for ADHD: an umbrella review. Lancet Psychiatry.

[7] Silk T, Dipnall L, Wong YT, Craig JM, Stanford SC, Sciberras E (2022). Epigenetics and ADHD. New discoveries in the behavioral neuroscience of attention-deficit hyperactivity disorder.

[8] Bartel DP (2009). MicroRNAs: target recognition and regulatory functions. Cell.

[9] Alural B, Genc S, Haggarty SJ (2017). Diagnostic and therapeutic potential of microRNAs in neuropsychiatric disorders: past, present, and future. Prog Neuropsychopharmacol Biol Psychiatry.

[10] Geaghan M, Cairns MJ (2015). MicroRNA and posttranscriptional dysregulation in psychiatry. Biol Psychiatry.

[11] Van Den Berg MMJ, Krauskopf J, Ramaekers JG, Kleinjans JCS, Prickaerts J, Briedé JJ (2020). Circulating microRNAs as potential biomarkers for psychiatric and neurodegenerative disorders. Prog Neurobiol.

[12] Cao P, Wang L, Cheng Q, Sun X, Kang Q, Dai L (2019). Changes in serum miRNA-let-7 level in children with attention deficit hyperactivity disorder treated by repetitive transcranial magnetic stimulation or atomoxetine: an exploratory trial. Psychiatry Res.

[13] Aydin SU, KabukcuBasay B, Cetin GO, Gungor Aydin A, Tepeli E (2019). Altered microRNA 5692b and microRNA let-7d expression levels in children and adolescents with attention deficit hyperactivity disorder. J Psychiatr Res.

[14] Sanchez-Mora C, Soler Artigas M, Garcia-Martinez I, Pagerols M, Rovira P, Richarte V (2019). Epigenetic signature for attention-deficit/hyperactivity disorder: identification of miR-26b-5p, miR-185-5p, and miR-191-5p as potential biomarkers in peripheral blood mononuclear cells. Neuropsychopharmacology.

[15] Nuzziello N, Craig F, Simone M, Consiglio A, Licciulli F, Margari L (2019). Integrated analysis of microRNA and mRNA expression profiles: an attempt to disentangle the complex interaction network in attention deficit hyperactivity disorder. Brain Sci.

[16] Wang LJ, Li SC, Kuo HC, Chou WJ, Lee MJ, Chou MC, et al. Gray matter volume and microRNA levels in patients with attention-deficit/hyperactivity disorder. Eur Arch Psychiatry Clin Neurosci. 2019;270:1037–45. 10.1007/s00406-019-01032-x 31240443

[17] Karadag M, Gokcen C, Nacarkahya G, Namiduru D, Dandil F, Calisgan B (2019). Chronotypical characteristics and related miR-142-3p levels of children with attention deficit and hyperactivity disorder. Psychiatry Res.

[18] Brum CB, Paixão-Côrtes VR, Carvalho AM, Martins-Silva T, Carpena MX, Ulguim KF, et al. Genetic variants in miRNAs differentially expressed during brain development and their relevance to psychiatric disorders susceptibility. World J Biol Psychiatry. 2020;22(6):456–67. 10.1080/15622975.2020.1834618 33040684

[19] Dypås L, Gutzkow K, Olsen A-K, Duale N. Molecular portrait of potential attention deficit/ hyperactivity disorder candidate genes and regulating Micrornas expression in normal human developing brain tissues. Med Res Arch. 2020;8(9). 10.18103/mra.v8i9.2214.

[20] Srivastav S, Walitza S, Grunblatt E (2018). Emerging role of miRNA in attention deficit hyperactivity disorder: a systematic review. Atten Defic Hyperact Disord.

[21] Kos MZ, Puppala S, Cruz D, Neary JL, Kumar A, Dalan E (2022). Blood-based miRNA biomarkers as correlates of brain-based miRNA expression. Front Mol Neurosci.

[22] Misiak B, Wójta-Kempa M, Samochowiec J, Schiweck C, Aichholzer M, Reif A (2022). Peripheral blood inflammatory markers in patients with attention deficit/hyperactivity disorder (ADHD): A systematic review and meta-analysis. Prog Neuropsychopharmacol Biol Psychiatry.

[23] Mowlem FD, Rosenqvist MA, Martin J, Lichtenstein P, Asherson P, Larsson H (2019). Sex differences in predicting ADHD clinical diagnosis and pharmacological treatment. Eur Child Adolesc Psychiatry.

[24] Sayal K, Prasad V, Daley D, Ford T, Coghill D (2018). ADHD in children and young people: prevalence, care pathways, and service provision. Lancet Psychiatry.

[25] Hanna J, Hossain GS, Kocerha J (2019). The Potential for microRNA therapeutics and clinical research. Front Genet.

[26] Maitre L, de Bont J, Casas M, Robinson O, Aasvang GM, Agier L (2018). Human Early Life Exposome (HELIX) study: a European population-based exposome cohort. BMJ Open.

[27] Wright J, Small N, Raynor P, Tuffnell D, Bhopal R, Cameron N (2013). Cohort profile: the born in Bradford multi-ethnic family cohort study. Int J Epidemiol.

[28] Heude B, Forhan A, Slama R, Douhaud L, Bedel S, Saurel-Cubizolles M-J (2016). Cohort profile: the EDEN mother-child cohort on the prenatal and early postnatal determinants of child health and development. Int J Epidemiol.

[29] Guxens M, Ballester F, Espada M, Fernández MF, Grimalt JO, Ibarluzea J (2012). Cohort profile: the INMA—INfancia y Medio Ambiente—(Environment and childhood) project. Int J Epidemiol.

[30] Grazuleviciene R, Danileviciute A, Nadisauskiene R, Vencloviene J (2009). Maternal smoking, GSTM1 and GSTT1 polymorphism and susceptibility to adverse pregnancy outcomes. Int J Environ Res Public Health.

[31] Magnus P, Birke C, Vejrup K, Haugan A, Alsaker E, Daltveit AK (2016). Cohort profile update: the Norwegian mother and child cohort study (MoBa). Int J Epidemiol.

[32] Chatzi L, Leventakou V, Vafeiadi M, Koutra K, Roumeliotaki T, Chalkiadaki G (2017). Cohort profile: the mother-child cohort in Crete, Greece (Rhea study). Int J Epidemiol.

[33] Forns J, Esnaola M, López-Vicente M, Suades-González E, Alvarez-Pedrerol M, Julvez J (2014). The n-back test and the attentional network task as measures of child neuropsychological development in epidemiological studies. Neuropsychology.

[34] Conners CK (1997). Conners’ rating scales - revised user’s manual.

[35] Achenbach T, Rescorla L (2001). Manual for the ASEBA school-age forms & profiles: an integrated system of multi-informant assessment Burlington, VT: University of Vermont. Research Center for Children, Youth, & Families.

[36] Duale N, Lipkin WI, Briese T, Aarem J, Ronningen KS, Aas KK (2014). Long-term storage of blood RNA collected in RNA stabilizing tempus tubes in a large biobank–evaluation of RNA quality and stability. BMC Res Notes.

[37] Suo C, Salim A, Chia KS, Pawitan Y, Calza S (2010). Modified least-variant set normalization for miRNA microarray. RNA.

[38] Ritchie ME, Phipson B, Wu D, Hu Y, Law CW, Shi W (2015). limma powers differential expression analyses for RNA-sequencing and microarray studies. Nucleic Acids Res.

[39] Leek JT, Storey JD (2007). Capturing heterogeneity in gene expression studies by surrogate variable analysis. PLoS Genet.

[40] Vives-Usano M, Hernandez-Ferrer C, Maitre L, Ruiz-Arenas C, Andrusaityte S, Borràs E (2020). In utero and childhood exposure to tobacco smoke and multi-layer molecular signatures in children. BMC Med.

[41] Boyce W, Torsheim T, Currie C, Zambon A (2006). The family affluence scale as a measure of national wealth: validation of an adolescent self-report measure. Soc Indic Res.

[42] Stekhoven DJ, Bühlmann P (2011). MissForest—non-parametric missing value imputation for mixed-type data. Bioinformatics.

[43] Licursi V, Conte F, Fiscon G, Paci P (2019). MIENTURNET: an interactive web tool for microRNA-target enrichment and network-based analysis. BMC Bioinformatics.

[44] Da Silveira W, Renaud L, Simpson J, Glen W, Hazard E, Chung D (2018). miRmapper: a tool for interpretation of miRNA–mRNA interaction networks. Genes.

[45] Vlachos IS, Zagganas K, Paraskevopoulou MD, Georgakilas G, Karagkouni D, Vergoulis T (2015). DIANA-miRPath v3.0: deciphering microRNA function with experimental support. Nucleic Acids Res.

[46] Dypås LB, Gützkow KB, Olsen A-K, Duale N (2020). MiRNA profiles in blood plasma from mother-child duos in human biobanks and the implication of sample quality: circulating miRNAs as potential early markers of child health. PLoS ONE.

[47] Coskun S, Karadag M, Gokcen C, Oztuzcu S (2021). miR-132 and miR-942 expression levels in children with attention deficit and hyperactivity disorder: a controlled study. Clin Psychopharmacol Neurosci.

[48] Wang LJ, Kuo HC, Lee SY, Huang LH, Lin Y, Lin PH (2022). MicroRNAs serve as prediction and treatment-response biomarkers of attention-deficit/hyperactivity disorder and promote the differentiation of neuronal cells by repressing the apoptosis pathway. Transl Psychiatry.

[49] Zhu P, Pan J, Cai QQ, Zhang F, Peng M, Fan XL (2022). MicroRNA profile as potential molecular signature for attention deficit hyperactivity disorder in children. Biomarkers.

[50] Kanehisa M, Goto S (2000). KEGG: kyoto encyclopedia of genes and genomes. Nucleic Acids Res.

[51] Kandemir H, Erdal ME, Selek S, Ay OI, Karababa IF, Kandemir SB (2014). Evaluation of several micro RNA (miRNA) levels in children and adolescents with attention deficit hyperactivity disorder. Neurosci Lett.

[52] Dobson-Patterson R, O’Gorman JG, Chan RCK, Shum DHK (2016). ADHD subtypes and neuropsychological performance in an adult sample. Res Dev Disabil.

[53] Grimm O, Weber H, Kittel-Schneider S, Kranz TM, Jacob CP, Lesch K-P, et al. Impulsivity and venturesomeness in an adult ADHD sample: relation to personality, comorbidity, and polygenic risk. Front Psychiatry. 2020;11:557160. 10.3389/fpsyt.2020.557160. 10.3389/fpsyt.2020.557160 PMC7768074 33381055

[54] Larsson H, Lichtenstein P, Larsson J-O (2006). Genetic contributions to the development of ADHD subtypes from childhood to adolescence. J Am Acad Child Adolesc Psychiatry.

[55] Willcutt EG (2012). The prevalence of DSM-IV attention-deficit/hyperactivity disorder: a meta-analytic review. Neurotherapeutics.

[56] Applegate B, Lahey BB, Hart EL, Biederman J, Hynd GW, Barkley RA (1997). Validity of the age-of-onset criterion for ADHD: a report from the DSM-IV field trials. J Am Acad Child Adolesc Psychiatry.

[57] Lahey BB, Applegate B, McBurnett K, Biederman J, Greenhill L, Hynd GW (1994). DSM-IV field trials for attention deficit hyperactivity disorder in children and adolescents. Am J Psychiatry.

[58] Barkley RA, Biederman J (1997). Toward a broader definition of the age-of-onset criterion for attention-deficit hyperactivity disorder. J Am Acad Child Adolesc Psychiatry.

[59] Young S, Adamo N, Ásgeirsdóttir BB, Branney P, Beckett M, Colley W (2020). Females with ADHD: an expert consensus statement taking a lifespan approach providing guidance for the identification and treatment of attention-deficit/ hyperactivity disorder in girls and women. BMC Psychiatry.

[60] Mowlem F, Agnew-Blais J, Taylor E, Asherson P (2019). Do different factors influence whether girls versus boys meet ADHD diagnostic criteria? Sex differences among children with high ADHD symptoms. Psychiatry Res.

[61] Wang LJ, Li SC, Lee MJ, Chou MC, Chou WJ, Lee SY (2018). Blood-Bourne MicroRNA biomarker evaluation in attention-deficit/hyperactivity disorder of Han Chinese individuals: an exploratory study. Front Psychiatry.

[62] Finnerty JR, Wang W-X, Hébert SS, Wilfred BR, Mao G, Nelson PT (2010). The miR-15/107 group of MicroRNA genes: evolutionary biology, cellular functions, and roles in human diseases. J Mol Biol.

[63] Foshay KM, Gallicano GI (2009). miR-17 family miRNAs are expressed during early mammalian development and regulate stem cell differentiation. Dev Biol.

[64] McGowan H, Mirabella VR, Hamod A, Karakhanyan A, Mlynaryk N, Moore JC (2018). hsa-let-7c miRNA regulates synaptic and neuronal function in human neurons. Front Synaptic Neurosci.

[65] Gururajan A, Naughton ME, Scott KA, O’Connor RM, Moloney G, Clarke G (2016). MicroRNAs as biomarkers for major depression: a role for let-7b and let-7c. Transl Psychiatry.

[66] Wu LH, Peng M, Yu M, Zhao QL, Li C, Jin YT (2015). Circulating MicroRNA Let-7d in attention-deficit/hyperactivity disorder. Neuromolecular Med.

[67] Indrieri A, Carrella S, Carotenuto P, Banfi S, Franco B (2020). The pervasive role of the miR-181 family in development, neurodegeneration, and cancer. Int J Mol Sci.

[68] Wan Y-Q, Feng J-G, Li M, Wang M-Z, Liu L, Liu X (2018). Prefrontal cortex miR-29b-3p plays a key role in the antidepressant-like effect of ketamine in rats. Exp Mol Med.

[69] Swahari V, Nakamura A, Hollville E, Stroud H, Simon JM, Ptacek TS (2021). MicroRNA-29 is an essential regulator of brain maturation through regulation of CH methylation. Cell Rep.

[70] Mogilyansky E, Rigoutsos I (2013). The miR-17/92 cluster: a comprehensive update on its genomics, genetics, functions and increasingly important and numerous roles in health and disease. Cell Death Differ.

[71] Kabekkodu SP, Shukla V, Varghese VK, D’ Souza J, Chakrabarty S, Satyamoorthy K (2018). Clustered miRNAs and their role in biological functions and diseases. Biol Rev.

[72] Doherty JL, Owen MJ (2014). Genomic insights into the overlap between psychiatric disorders: implications for research and clinical practice. Genome Med.

[73] Gudmundsson OO, Walters GB, Ingason A, Johansson S, Zayats T, Athanasiu L (2019). Attention-deficit hyperactivity disorder shares copy number variant risk with schizophrenia and autism spectrum disorder. Transl Psychiatry.

[74] Zablotsky B, Bramlett MD, Blumberg SJ (2020). The co-occurrence of autism spectrum disorder in children with ADHD. J Atten Disord.

[75] Salazar F, Baird G, Chandler S, Tseng E, O’Sullivan T, Howlin P (2015). Co-occurring psychiatric disorders in preschool and elementary school-aged children with autism spectrum disorder. J Autism Dev Disord.

