# Supplementary material for: Blood miRNA levels associated with ADHD traits in children across six European birth cohorts
Source: BMC Psychiatry. 2023 Sep 25;23:696. doi: 10.1186/s12888-023-05199-5 (PMC10521440; doi:10.1186/s12888-023-05199-5)
Supplement: Supplementary file 1 — Additional file 1. Additional details of methods. [file 12888_2023_5199_MOESM1_ESM.pdf]

Additional file 1: Additional details of methods

RNA extraction and quality control

RNA was extracted from 1,690 HELIX whole blood samples using the MagMAX for Stabilized Blood Tubes RNA Isolation Kit (Thermo Fisher Scientific, USA). Samples were processed by arrival, i.e., one cohort at a time. RNA was extracted in two main rounds: 1,382 samples in the first round, and 308 extra samples in the second round. The quality of RNA was evaluated with a 2100 Bioanalyzer (Agilent Technologies, USA) and the concentration with a NanoDrop 1000 UV-Vis Spectrophotometer. We obtained 1,304 samples with good RNA quality (1,087 in the first round and 217 in the second round). Samples classified as good RNA quality had a RIN >5, a similar RNA integrity pattern in the visual inspection and a concentration >10 ng/ul. Mean values for the RIN, concentration (ng/ul) and Nanodrop 260/230 ratio were: 7.05, 109.07 and 2.15.

miRNA laboratory processing

miRNA quantification was done in 2 rounds with the SurePrint Human miRNA Microarray rel. 21 (Agilent Technologies, USA) at the Genomics Core Facility at the Centre for Genomic Regulation (CRG, Spain). In the first round, 1,126 samples (1,087 unique HELIX samples and 39 controls - a mixture of RNAs from several human tissues: universal miRNA reference kit) were processed; while in the second round, 216 samples (207 HELIX samples, 180 unique subjects, including inter-round and intra-round duplicates, and 9 controls). Samples were randomized by sex and cohort. Batches consisted of 24 samples which were hybridized onto 3 slides (8 samples per slide). Inter-round (N=18) and intra-round (N=9) duplicates of the second round were distributed across batches and intra-round duplicates were not placed in the same array. Sample input was 100 ng of total RNA. Samples were processed following Agilent's recommendations. Briefly, RNA samples were concentrated or evaporated to reach the required concentration using SpeedVac. Raw data was extracted with the Agilent Feature Extraction software. Samples that did not pass the laboratory quality control parameters were repeated (N=52 in the first round and N=10 in the second round). miRNAs were annotated with the Annotation_70156 version from Agilent and with additional information from mirBase v21 (http://www.mirbase.org/).

Quality control, normalization, and batch correction

Samples with low quality were filtered based on several Agilent QC parameters and calculated sample call rate. The average number of detected miRNAs per sample was higher in the first round compared with the second round. miRNAs were considered not detected when the expression signal was no different from the background, or the standard error of the different probes was >3 times higher than the expression signal.

miRNA expression levels were normalized using the least variant set (LVS) method (1), with background correction by the Normexp method in the limma R package (2). The LVS method identifies a subset of miRNAs with the smallest array-to-array variation which then are used to normalize the miRNA expression values among samples. For the identification of housekeeping miRNAs, a random sample of 50 HELIX samples was used. miRNA expression values were $\log_2$ transformed.

Technical batch effects were investigated using Principal Component Analysis (PCA). We found that round, slide, and cohort were the main drivers of the structure of the data. To eliminate these batch effects, we tested the ComBat (3) and the Surrogate Variable Analysis (SVA) (4) methods. In the case of ComBat we selected the slide effect as the batch variable to be controlled; and for SVA we obtained the residuals of surrogate variables (SVs) calculated protecting cohort, sex and age. Performance of ComBat and SVA were inspected by PCA and by inter- and intra-round duplicate samples agreement calculating the Concordance Correlation Coefficient (CCC). We found that SVA outperformed ComBat at different miRNA call rate thresholds, and thus the residuals of SVs were used to test their association with our chosen measures of behavior and neuropsychological functions. For the N-back test 23 SVs were calculated, while 25 SVs were calculated for all other measures.

Finally, inter- and intra-round duplicates and 5 outliers in the PCA after batch correction were eliminated from the dataset. We only kept autosomal miRNAs with a call rate >70%. The final dataset consisted of 1126 unique HELIX samples (952 in round 1 and 174 in round 2) and 308 autosomal miRNAs.

Literature search

A literature search was performed in PubMed August 31st, 2022, with the search terms below.

Search: ((attention AND deficit AND hyperactivity AND disorder) OR adhd OR (attention AND deficit AND disorder) OR (hyperactivity AND disorder) OR (hyperkinetic AND disorder) OR (hyperactive AND disorder) OR (attention AND deficit)) AND ((noncoding AND (rna OR rnas)) OR (non-coding AND (rna OR rnas)) OR (untranslated AND (rna OR rnas)) OR microrna OR micrornas OR mirna OR mirnas)

References

1. Suo C, Salim A, Chia KS, Pawitan Y, Calza S. Modified least-variant set normalization for miRNA microarray. RNA. 2010;16(12):2293-303.
2. Ritchie ME, Phipson B, Wu D, Hu Y, Law CW, Shi W, et al. limma powers differential expression analyses for RNA-sequencing and microarray studies. Nucleic Acids Res. 2015;43(7):e47-e.
3. Johnson WE, Li C, Rabinovic A. Adjusting batch effects in microarray expression data using empirical Bayes methods. Biostatistics. 2007;8(1):118-27.
4. Leek JT, Storey JD. Capturing heterogeneity in gene expression studies by surrogate variable analysis. PLoS Genet. 2007;3(9):1724-35.
