# Supplementary material for: Blood miRNA levels associated with ADHD traits in children across six European birth cohorts
Source: BMC Psychiatry. 2023 Sep 25;23:696. doi: 10.1186/s12888-023-05199-5 (PMC10521440; doi:10.1186/s12888-023-05199-5)
Supplement: Supplementary file 2 — Additional file 2: Fig. S1. PCA (principal component analysis) before and after SVA (surrogate variable analysis). Fig. S2. Correlation matrix for bivariate association between measures of behavior and neuropsychological functions and child’s age. Fig. S3. Behavioral and neuropsychological scores by sex. [file 12888_2023_5199_MOESM2_ESM.pdf]

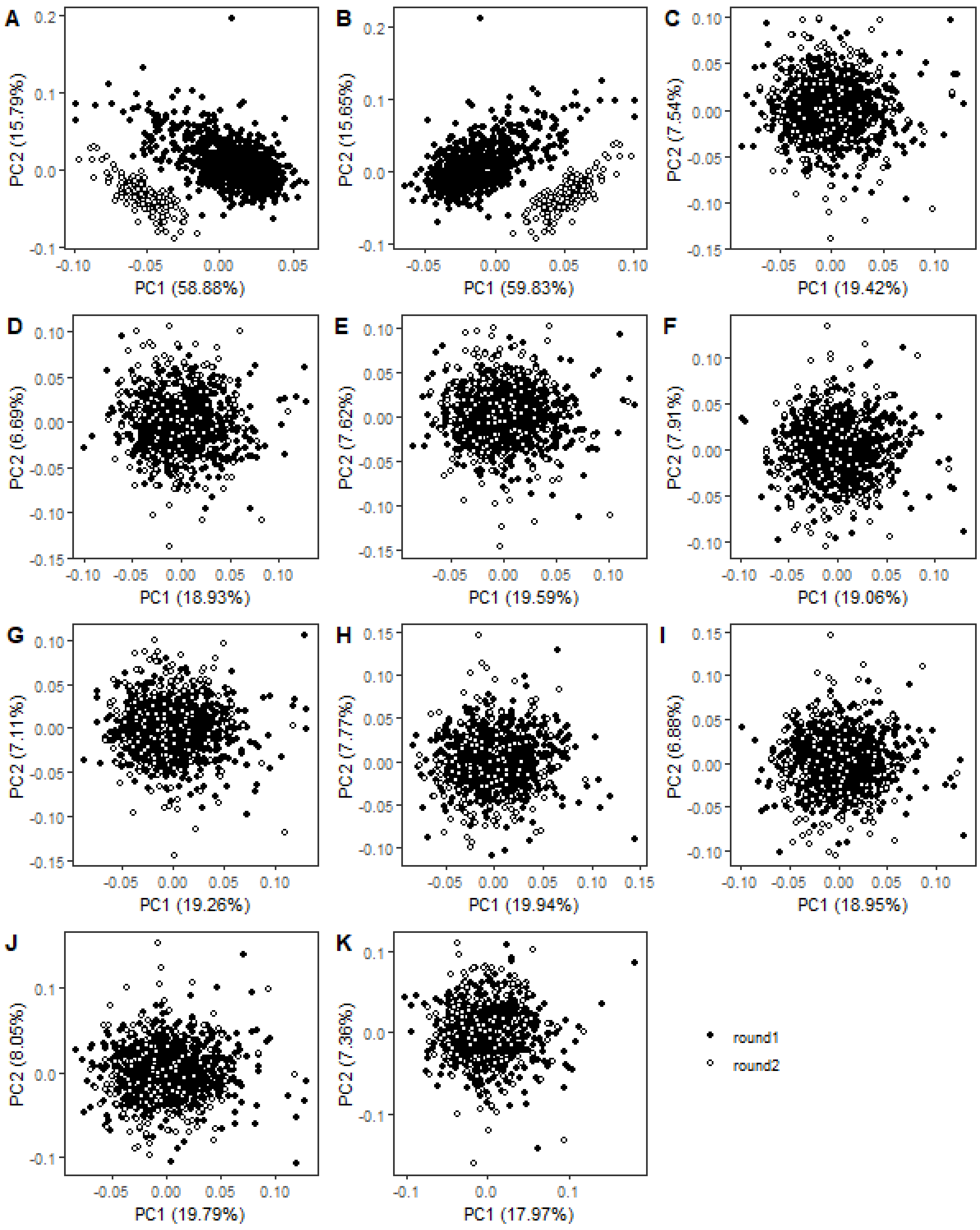

Fig. S1. PCA (principal component analysis) before and after SVA (surrogate variable analysis). Showing effect of SVA correction on laboratory processing round. PCA before SVA for **(A)** dataset N=1089 and **(B)** dataset N=894. PCA after SVA for **(C)** Conner's ADHD index, **(D)** Conner's hyperactivity, **(E)** Conner's inattention, **(F)** CBCL ADHD problems, **(G)** ANT HRT-SE, **(H)** ANT zeros, **(I)** ANT omissions, **(J)** ANT conflict, and **(K)** N-back *d*'.

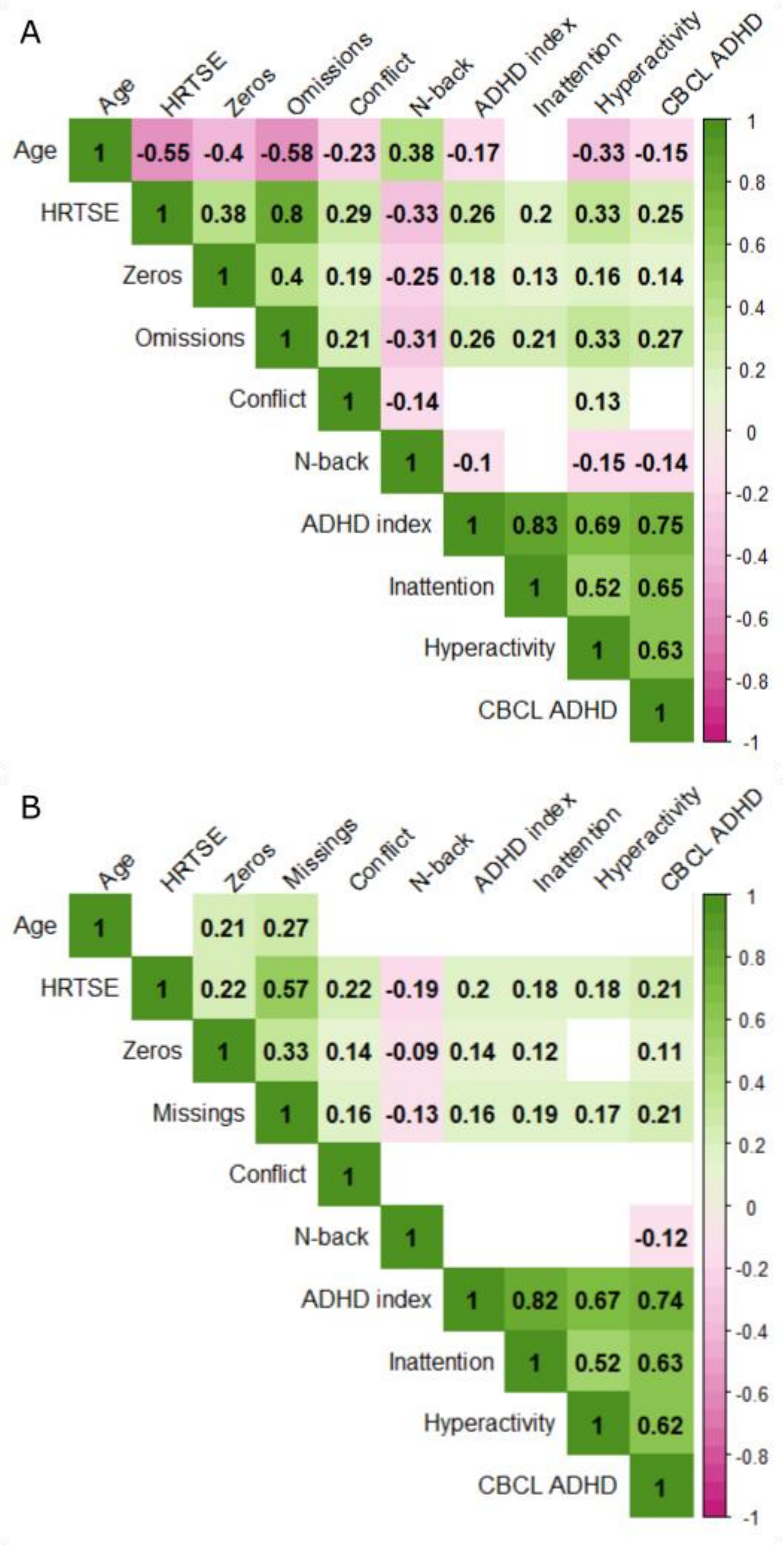

Fig. S2. Correlation matrix for bivariate association between measures of behavior and neuropsychological functions and child's age. Spearman rank correlation coefficient is presented. Analysis performed **(A)** prior to, and **(B)** after, age-adjusting and transforming measures of behavior and neuropsychological functions. A blank square indicates no statistical significance, with a significance level of 0.01. Positive correlations are green and negative correlations are pink, and the intensity of colors is proportional with the correlation coefficient.

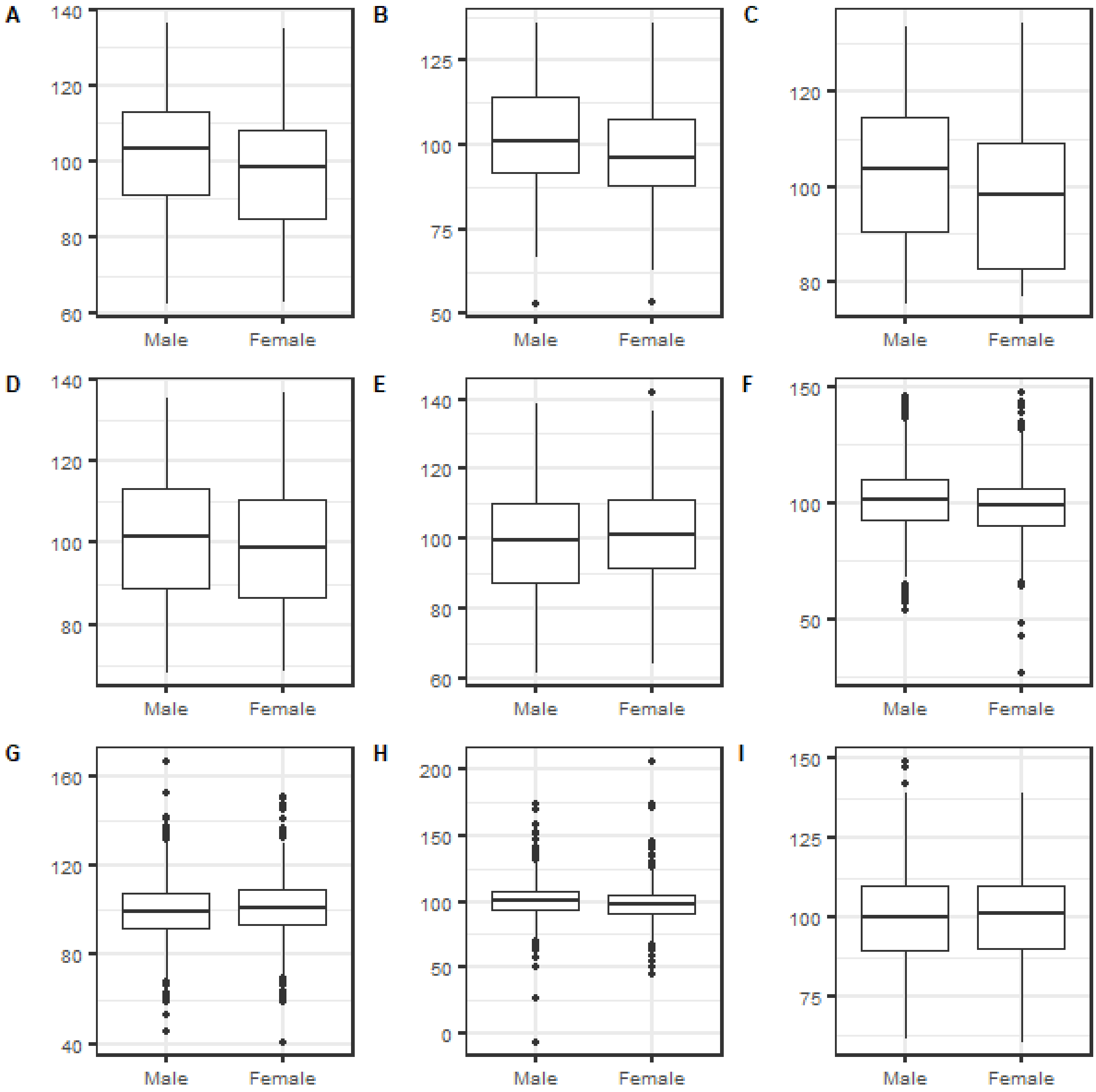

Fig. S3. Behavioral and neuropsychological scores by sex. (A) Conner's ADHD index, **(B)** Conner's hyperactivity, **(C)** Conner's inattention, **(D)** CBCL ADHD problems, **(E)** ANT HRT-SE, **(F)** ANT zeros, **(G)** ANT omissions, **(H)** ANT conflict, and **(I)** N-back *d'*.
