# Supplementary material for: Blood miRNA levels associated with ADHD traits in children across six European birth cohorts
Source: BMC Psychiatry. 2023 Sep 25;23:696. doi: 10.1186/s12888-023-05199-5 (PMC10521440; doi:10.1186/s12888-023-05199-5)
Supplement: Supplementary file 3 — Additional file 3: Table S1. Change in number of miRNAs significantly differentially expressed after sensitivity analyses. Table S2. miRNAs linked to ADHD in previous literature. Table S3. Functional enrichment analysis of 29 miRNAs significantly associated with hyperactivity. Table S4. Functional enrichment analysis of 15 miRNAs associated with hyperactivity/ADHD in this and previous studies. Table S5. MiRNAs statistically significant for Conner’s hyperactivity or inattention score, and their previous associations with neurodevelopmental or psychiatric phenotypes. [file 12888_2023_5199_MOESM3_ESM.pdf]

Table S1. Change in number of miRNAs significantly differentially expressed after sensitivity analyses.

		ANT				N-back	Conner's			CBCL	New significant miRNAs
		**HRT-SE**	**Zeros**	**Omissions**	**Conflict**	**3 numbers**	**ADHD Index**	**Inattention**	**Hyperactivity**	**ADHD**	
**Sex**	Each separately:										
	**Female**							0	0		
	**Male**							1D, 1UP	4D		
**SES**	(as covariate)							1D, 2UP	26D		
**Cohort**	One removed:										
	**BIB**							0	15D		Hyper.: miR-378a-3p
	**EDEN**							1D	28D		Hyper.: miR-126-3p, 425-5p, 15a-5p, 199a-3p
	**INMA**				1D			0	0		Conflict: miR-6812-5p
	**KANC**							1D, 5UP	16D	3UP	Inatt.: miR-4687-3p, 4788, 7152-3p<br>CBCL: miR-1268b, 4763-3p, 1268a
	**MOBA**							0	25D		
	**RHEA**							1D, 2UP	0		
**Cohort**	Each separately:										
	**BIB**							0	0		
	**EDEN**							0	0		
	**INMA**							0	7D		Hyper.: miR-378a-3p
	**KANC**						2UP	0	0		ADHD I.: miR-4749-3p, 6875-5p
	**MOBA**							0	0		
	**RHEA**							0	0		
**Round**	(as covariate)							1D, 2UP	28D		
**Round**	Each separately:										
	**Round 1**						1UP	1UP	0		ADHD I.: miR-4443
	**Round 2**							0	0		

D, downregulated; UP, upregulated.

Table S2. miRNAs linked to ADHD in previous literature.

miRNA	Status in ADHD	Sample type	References
hsa-let-7d	Upregulated (two studies)	Serum	(1, 2)
hsa-let-7d	Downregulated	WB	(3)
hsa-let-7g-5p	ADHD-prediction model	WBCs	(4)
hsa-let-7g-5p	Decreased in patient group responding to MPH treatment	WBCs	(5)
hsa-miR-101-3p	ADHD-prediction model	WBCs	(4)
hsa-miR-101-3p	Upregulated	Serum	(6)
hsa-miR-101-3p	Decreased in both responder and non-responder patient group after MPH treatment	WBCs	(5)
hsa-miR-106b-5p	Downregulated	Serum	(6)
hsa-miR-106b-5p	Downregulated	WB	(7)
hsa-miR-107	Downregulated	WB	(7)
hsa-miR-124-3p	Targeting ADHD gene set	Predicted	(8)
hsa-miR-125a-3p	Targeting ADHD gene set	Predicted	(8)
hsa-miR-126-5p	ADHD-prediction model	WBCs	(4)
hsa-miR-126-5p	Upregulated	WBCs	(9)
hsa-miR-126-5p	Decreased in patient group responding to MPH treatment	WBCs	(5)
hsa-miR-129-5p	Targeting ADHD gene set	Predicted	(8)
hsa-miR-130a-3p	Upregulated	Serum	(6)
hsa-miR-132-3p	Upregulated	Plasma	(10)
hsa-miR-138-5p	Upregulated	Serum	(6)
hsa-miR-1-3p	Targeting ADHD gene set	Predicted	(8)
hsa-miR-140-3p	ADHD-prediction model	WBCs	(4)
hsa-miR-140-3p	Upregulated	WBCs	(9)
hsa-miR-140-3p	Decreased in patient group responding to MPH treatment	WBCs	(5)
hsa-miR-142-3p	Downregulated in ADHD group when presence of psychiatric disease in their families.	Plasma	(11)
hsa-miR-142-5p	ADHD-prediction model	WBCs	(4)
hsa-miR-148b-3p	Downregulated	WB	(12)
hsa-miR-150-5p	ADHD-prediction model	WBCs	(4)
hsa-miR-150-5p	Increased in patient group not responding to MPH treatment	WBCs	(5)
hsa-miR-151a-3p	ADHD-prediction model	WBCs	(4)
hsa-miR-151a-3p	Decreased in patient group responding to MPH treatment	WBCs	(5)
hsa-miR-151a-5p	ADHD-prediction model	WBCs	(4)

hsa-miR-151a-5p	Decreased in patient group responding to MPH treatment	WBCs	(5)
hsa-miR-155-5p	Targeting ADHD gene set	Predicted	(8)
hsa-miR-155-5p	Upregulated	WB	(7)
hsa-miR-15a-5p	Targeting ADHD gene set	Predicted	(8)
hsa-miR-16-5p	Targeting ADHD gene set	Predicted	(8)
hsa-miR-17-5p	Targeting ADHD gene set	Predicted	(8)
hsa-miR-185-5p	Downregulated	PBMCs	(13)
hsa-miR-18a-5p	Downregulated	WB	(7)
hsa-miR-191-5p	Upregulated	PBMCs	(13)
hsa-miR-195-5p	Upregulated	Serum	(6)
hsa-miR-19a-3p	Targeting ADHD gene set	Predicted	(8)
hsa-miR-19b-3p	Targeting ADHD gene set	Predicted	(8)
hsa-miR-204-5p	Targeting ADHD gene set	Predicted	(8)
hsa-miR-21-5p	Targeting ADHD gene set	Predicted	(8)
hsa-miR-223-3p	ADHD-prediction model	WBCs	(4)
hsa-miR-22-3p	Downregulated	WB	(7)
hsa-miR-22-3p	Targeting ADHD gene set	Predicted	(8)
hsa-miR-24-3p	Downregulated	WB	(7)
hsa-miR-24-3p	Targeting ADHD gene set	Predicted	(8)
hsa-miR-26b-5p	Downregulated	PBMCs	(13)
hsa-miR-27a-3p	ADHD-prediction model	WBCs	(4)
hsa-miR-27a-3p	Decreased in patient group responding to MPH treatment	WBCs	(5)
hsa-miR-30a-5p	Targeting ADHD gene set	Predicted	(8)
hsa-miR-30e-5p	ADHD-prediction model	WBCs	(4)
hsa-miR-30e-5p	Upregulated	WBCs	(9)
hsa-miR-30e-5p	Decreased in patient group responding to MPH treatment	WBCs	(5)
hsa-miR-335-5p	Targeting ADHD gene set	Predicted	(8)
hsa-miR-34a-5p	Targeting ADHD gene set	Predicted	(8)
hsa-miR-34c-3p	Upregulated	PBMCs	(14)
hsa-miR-378a-5p	Downregulated in ADHD group when presence of psychiatric disease in their families.	Plasma	(11)
hsa-miR-4281	ADHD-prediction model + downregulated. Increased after 12-week MPH treatment.	Serum	(15)
hsa-miR-4466	ADHD-prediction model + upregulated. Decreased after 12-week MPH treatment.	Serum	(15)
hsa-miR-4516	ADHD-prediction model + downregulated. Increased after 12-week MPH treatment.	Serum	(15)

hsa-miR-4763-3p	ADHD-prediction model + downregulated. Increased after 12-week MPH treatment.	Serum	(15)
hsa-miR-486-5p	ADHD-prediction model	WBCs	(4)
hsa-miR-486-5p	Decreased in patient group responding to MPH treatment	WBCs	(5)
hsa-miR-5692b	Upregulated	WB	(3)
hsa-miR-6090	ADHD-prediction model + downregulated. Increased after 12-week MPH treatment.	Serum	(15)
hsa-miR-616-5p	Targeting ADHD gene set	Predicted	(8)
hsa-miR-652-3p	Upregulated	WB	(12)
hsa-miR-92a-3p	ADHD-prediction model	WBCs	(4)
hsa-miR-942-5p	Upregulated	WB	(12)
hsa-miR-9-5p	Targeting ADHD gene set	Predicted	(8)
hsa-miR-96-5p	Targeting ADHD gene set	Predicted	(8)

WB, whole blood; WBCs, white blood cells; PBMCs, peripheral blood mononuclear cells; MPH, methylphenidate.

Table S3. Functional enrichment analysis of 29 miRNAs significantly associated with hyperactivity.

KEGG pathway	#miRNAs	#genes	*p* value
Proteoglycans in cancer	27	159	5.36e-22
Viral carcinogenesis	27	157	6.46e-15
Renal cell carcinoma	27	60	2.71e-11
Hippo signaling pathway	27	114	4.86e-11
Protein processing in endoplasmic reticulum	27	128	7.47e-09
ECM-receptor interaction	25	51	1.11e-07
Cell cycle	27	97	1.11e-07
Ubiquitin mediated proteolysis	27	109	2.55e-07
Endocytosis	27	152	2.92e-07
TGF-beta signaling pathway	27	61	3.32e-07
Adherens junction	27	61	4.08e-07
Estrogen signaling pathway	27	74	5.94e-07
Hepatitis B	27	104	6.86e-07
Glioma	26	51	7.97e-07
Prostate cancer	27	72	1.35e-06
Chronic myeloid leukemia	26	60	2.07e-06
Fatty acid metabolism	22	33	2.99e-06
Thyroid hormone signaling pathway	27	88	2.99e-06
Signaling pathways regulating pluripotency of stem cells	27	103	3.01e-06
Pathways in cancer	27	268	3.58e-06
Oocyte meiosis	27	82	8.49e-06
Colorectal cancer	27	51	1.06e-05
Glycosaminoglycan biosynthesis - keratan sulfate	19	12	1.26e-05
p53 signaling pathway	27	54	2.38e-05
Pancreatic cancer	26	52	2.69e-05
Bacterial invasion of epithelial cells	26	59	2.82e-05
N-Glycan biosynthesis	25	37	3.60e-05

FoxO signaling pathway	27	98	3.73e-05
AMPK signaling pathway	27	93	5.67e-05
Prion diseases	25	21	9.46e-05
Bladder cancer	26	33	9.60e-05
Focal adhesion	27	144	1.23e-04
Sphingolipid signaling pathway	26	84	1.24e-04
Small cell lung cancer	27	66	1.26e-04
Endometrial cancer	26	42	1.31e-04
Thyroid cancer	25	24	1.33e-04
Non-small cell lung cancer	25	43	2.35e-04
Epstein-Barr virus infection	27	140	2.94e-04
Lysine degradation	26	34	3.04e-04
ErbB signaling pathway	27	64	3.28e-04
mTOR signaling pathway	27	48	4.31e-04
HIF-1 signaling pathway	27	79	4.58e-04
Other types of O-glycan biosynthesis	24	20	6.03e-04
Transcriptional misregulation in cancer	27	116	8.72e-04
Progesterone-mediated oocyte maturation	25	65	1.50e-03
Toxoplasmosis	27	84	1.60e-03
HTLV-I infection	27	172	1.60e-03
Central carbon metabolism in cancer	27	51	1.67e-03
Regulation of actin cytoskeleton	27	139	1.70e-03
TNF signaling pathway	26	79	1.76e-03
Spliceosome	27	92	1.97e-03
Neurotrophin signaling pathway	27	84	2.80e-03
Chagas disease (American trypanosomiasis)	27	70	3.84e-03
RNA transport	27	112	4.49e-03
Axon guidance	27	85	4.49e-03
Acute myeloid leukemia	26	43	4.87e-03
Insulin signaling pathway	27	96	4.95e-03

Fatty acid degradation	21	25	5.18e-03
Prolactin signaling pathway	26	51	5.24e-03
Wnt signaling pathway	26	91	5.25e-03
Fatty acid elongation	17	13	5.80e-03
Shigellosis	25	46	6.33e-03
Gap junction	26	58	7.53e-03
Steroid biosynthesis	19	14	7.74e-03
Pathogenic Escherichia coli infection	26	41	8.92e-03
Inositol phosphate metabolism	27	42	8.93e-03
Melanoma	27	49	8.93e-03
RNA degradation	26	54	9.03e-03
Glycosaminoglycan biosynthesis - heparan sulfate / heparin	22	17	1.16e-02
mRNA surveillance pathway	27	65	2.97e-02
Platelet activation	26	82	3.03e-02
Terpenoid backbone biosynthesis	21	16	3.19e-02
Fatty acid biosynthesis	22	7	3.44e-02
Apoptosis	27	59	4.02e-02
Lysosome	25	78	4.53e-02
Adrenergic signaling in cardiomyocytes	27	85	4.92e-02

Table S4. Functional enrichment analysis of 15 miRNAs associated with hyperactivity/ADHD in this and previous studies.

KEGG pathway	#miRNAs	#genes	*p* value
Proteoglycans in cancer	14	149	1.08e-26
Viral carcinogenesis	14	140	1.08e-12
Hippo signaling pathway	14	106	2.08e-12
Protein processing in endoplasmic reticulum	14	118	3.76e-09
Endocytosis	14	141	7.57e-08
Signaling pathways regulating pluripotency of stem cells	14	98	1.47e-07
Glioma	13	48	2.53e-07
Adherens junction	14	55	2.53e-07
Renal cell carcinoma	14	53	3.52e-07
TGF-beta signaling pathway	14	56	3.66e-07
Cell cycle	14	89	4.72e-07
Ubiquitin mediated proteolysis	14	99	6.56e-07
Thyroid hormone signaling pathway	14	82	1.04e-06
AMPK signaling pathway	14	89	2.16e-06
Bacterial invasion of epithelial cells	14	57	2.68e-06
Chronic myeloid leukemia	14	55	3.45e-06
Pancreatic cancer	14	49	4.45e-06
Non-small cell lung cancer	13	42	1.19e-05
Pathways in cancer	14	240	1.60e-05
Sphingolipid signaling pathway	14	79	1.72e-05
Hepatitis B	14	91	1.96e-05
Bladder cancer	14	32	1.96e-05
Oocyte meiosis	14	77	2.23e-05
Prion diseases	13	18	2.66e-05
mTOR signaling pathway	14	47	2.91e-05
FoxO signaling pathway	14	90	3.41e-05
N-Glycan biosynthesis	14	34	4.26e-05

Estrogen signaling pathway	14	67	4.26e-05
Prostate cancer	14	63	4.33e-05
Glycosaminoglycan biosynthesis - keratan sulfate	12	10	4.36e-05
p53 signaling pathway	14	50	4.36e-05
Endometrial cancer	13	39	1.10e-04
Central carbon metabolism in cancer	14	48	1.33e-04
Insulin signaling pathway	14	93	1.64e-04
Colorectal cancer	14	44	1.74e-04
Lysine degradation	14	32	2.03e-04
RNA transport	14	106	2.96e-04
Focal adhesion	14	129	3.27e-04
Thyroid cancer	13	22	4.61e-04
Small cell lung cancer	14	59	5.61e-04
Steroid biosynthesis	11	14	7.23e-04
ECM-receptor interaction	14	45	7.23e-04
Other types of O-glycan biosynthesis	12	19	7.29e-04
Progesterone-mediated oocyte maturation	13	60	9.75e-04
Shigellosis	13	44	9.93e-04
HIF-1 signaling pathway	14	71	1.00e-03
Fatty acid elongation	10	11	1.19e-03
Prolactin signaling pathway	13	47	1.19e-03
Fatty acid metabolism	13	29	1.54e-03
TNF signaling pathway	13	72	1.54e-03
Gap junction	14	54	1.72e-03
Spliceosome	14	82	2.06e-03
Fatty acid biosynthesis	13	7	2.49e-03
Pathogenic Escherichia coli infection	14	39	2.97e-03
RNA degradation	14	51	4.24e-03
ErbB signaling pathway	14	56	4.37e-03
Neurotrophin signaling pathway	14	76	4.54e-03

Regulation of actin cytoskeleton	14	123	4.66e-03
Axon guidance	14	78	5.30e-03
Chagas disease (American trypanosomiasis)	14	63	5.30e-03
Melanoma	14	45	5.33e-03
Terpenoid backbone biosynthesis	12	16	5.35e-03
Wnt signaling pathway	14	81	5.53e-03
Transcriptional misregulation in cancer	14	100	6.98e-03
mRNA surveillance pathway	14	61	7.36e-03
Glycosaminoglycan biosynthesis - heparan sulfate / heparin	14	15	9.11e-03
HTLV-I infection	14	150	1.27e-02
Lysosome	14	73	1.49e-02
Adrenergic signaling in cardiomyocytes	14	76	1.49e-02
Platelet activation	13	75	1.69e-02
VEGF signaling pathway	13	40	1.92e-02
Inositol phosphate metabolism	14	38	1.92e-02
Acute myeloid leukemia	13	37	2.09e-02
Circadian rhythm	13	22	2.54e-02
Epstein-Barr virus infection	14	117	2.84e-02
Toxoplasmosis	14	70	3.50e-02
Non-alcoholic fatty liver disease (NAFLD)	14	85	4.23e-02
Vitamin B6 metabolism	3	5	4.57e-02
Vasopressin-regulated water reabsorption	14	32	4.82e-02

Table S5. MiRNAs statistically significant for Conner's hyperactivity or inattention score, and their previous associations with neurodevelopmental or psychiatric phenotypes.

ADHD-trait	miRNA	ADHD	ASD	SZ	MDD	BD	References
**Hyperactivity**							
	hsa-let-7c-5p				✓		MDD (16)
	hsa-let-7i-5p						
	hsa-miR-101-3p	✓	✓		✓		ADHD (4, 6), ASD (17), MDD (18, 19)
	hsa-miR-103a-3p		✓				ASD (20)
	hsa-miR-106b-5p	✓	✓	✓		✓	ADHD (6, 7), ASD (21), SZ (22, 23), BD (24, 25)
	hsa-miR-107	✓	✓	✓	✓	✓	ADHD (7), ASD (26), SZ (24), MDD (24), BD (25)
	hsa-miR-130a-3p	✓	✓	✓			ADHD (6), ASD (17), SZ (22)
	hsa-miR-140-3p	✓	✓			✓	ADHD (4, 9), ASD (27), BD (19)
	hsa-miR-142-3p	✓	✓		✓	✓	ADHD (11), ASD (26), MDD (24), BD (28)
	hsa-miR-144-3p		✓				ASD (29)
	hsa-miR-146b-5p		✓	✓			ASD (26, 30), SZ (31)
	hsa-miR-148b-3p	✓	✓	✓			ADHD (12), ASD (26), SZ (22)
	hsa-miR-150-5p	✓	✓	✓			ADHD (4), ASD (32), SZ (24)
	hsa-miR-181a-5p		✓	✓			ASD (26), SZ (22)
	hsa-miR-181b-5p		✓	✓			ASD (26), SZ (19, 33, 34)
	hsa-miR-186-5p		✓	✓		✓	ASD (26), SZ (35), BD (35)
	hsa-miR-19a-3p	✓	✓		✓		ADHD (8), ASD (26, 32), MDD (36)
	hsa-miR-19b-3p	✓	✓	✓	✓	✓	ADHD (8), ASD (26), SZ (37), MDD (38), BD (39)
	hsa-miR-24-3p	✓		✓	✓		ADHD (7, 8), SZ (22), MDD (19)
	hsa-miR-27a-3p	✓	✓	✓			ADHD (4), ASD (26), SZ (22)
	hsa-miR-29a-3p		✓	✓		✓	ASD (40), SZ (41), BD (24, 25)
	hsa-miR-29b-3p		✓	✓			ASD (26), SZ (22)
	hsa-miR-29c-3p			✓		✓	SZ (24), BD (24)
	hsa-miR-30e-5p	✓	✓	✓			ADHD (4, 9), ASD (21), SZ (19)
	hsa-miR-324-5p						

	hsa-miR-342-5p				✓	MDD (19)
	hsa-miR-378i					
	hsa-miR-92a-3p	✓	✓	✓	✓	ADHD (4), ASD (26), SZ (42, 43), MDD (19)
	hsa-miR-942-5p	✓				ADHD (12)
**Inattention**						
	hsa-miR-3180-3p					
	hsa-miR-4257					
	hsa-miR-4443		✓			ASD (26)

Not an exhaustive list. ADHD, attention-deficit/hyperactivity disorder; ASD, autism spectrum disorder; SZ, schizophrenia; MDD, major depression disorder; BD: bipolar disorder.

References

1. Cao P, Wang L, Cheng Q, Sun X, Kang Q, Dai L, et al. Changes in serum miRNA-let-7 level in children with attention deficit hyperactivity disorder treated by repetitive transcranial magnetic stimulation or atomoxetine: An exploratory trial. Psychiatry Res. 2019;274:189-94.
2. Wu LH, Peng M, Yu M, Zhao QL, Li C, Jin YT, et al. Circulating MicroRNA Let-7d in Attention-Deficit/Hyperactivity Disorder. Neuromolecular Med. 2015;17(2):137-46.
3. Aydin SU, Kabukcu Basay B, Cetin GO, Gungor Aydin A, Tepeli E. Altered microRNA 5692b and microRNA let-7d expression levels in children and adolescents with attention deficit hyperactivity disorder. J Psychiatr Res. 2019;115:158-64.
4. Wang LJ, Li SC, Lee MJ, Chou MC, Chou WJ, Lee SY, et al. Blood-Bourne MicroRNA Biomarker Evaluation in Attention-Deficit/Hyperactivity Disorder of Han Chinese Individuals: An Exploratory Study. Front Psychiatry. 2018;9:227.
5. Wang L-J, Kuo H-C, Lee S-Y, Huang L-H, Lin Y, Lin P-H, et al. MicroRNAs serve as prediction and treatment-response biomarkers of attention-deficit/hyperactivity disorder and promote the differentiation of neuronal cells by repressing the apoptosis pathway. Transl Psychiatry. 2022;12(1).
6. Zadehbagheri F, Hosseini E, Bagheri-Hosseinabadi Z, Rekabdarkolaee HM, Sadeghi I. Profiling of miRNAs in serum of children with attention-deficit hyperactivity disorder shows significant alterations. J Psychiatr Res. 2019;109:185-92.
7. Kandemir H, Erdal ME, Selek S, Ay OI, Karababa IF, Kandemir SB, et al. Evaluation of several micro RNA (miRNA) levels in children and adolescents with attention deficit hyperactivity disorder. Neurosci Lett. 2014;580:158-62.
8. Dypås L, Gutzkow K, Olsen A-K, Duale N. Molecular Portrait of Potential Attention Deficit/ Hyperactivity Disorder Candidate Genes and Regulating Micrornas Expression in Normal Human Developing Brain Tissues. Med Res Arch. 2020;8(9).
9. Wang LJ, Li SC, Kuo HC, Chou WJ, Lee MJ, Chou MC, et al. Gray matter volume and microRNA levels in patients with attention-deficit/hyperactivity disorder. Eur Arch Psychiatry Clin Neurosci. 2019.

10. Coskun S, Karadag M, Gokcen C, Oztuzcu S. miR-132 and miR-942 Expression Levels in Children with Attention Deficit and Hyperactivity Disorder: A Controlled Study. Clin Psychopharmacol Neurosci. 2021;19(2):262-8.
11. Karadag M, Gokcen C, Nacarkahya G, Namiduru D, Dandil F, Calisgan B, et al. Chronotypical characteristics and related miR-142-3p levels of children with attention deficit and hyperactivity disorder. Psychiatry Res. 2019;273:235-9.
12. Nuzziello N, Craig F, Simone M, Consiglio A, Licciulli F, Margari L, et al. Integrated Analysis of microRNA and mRNA Expression Profiles: An Attempt to Disentangle the Complex Interaction Network in Attention Deficit Hyperactivity Disorder. Brain Sci. 2019;9(10).
13. Sanchez-Mora C, Soler Artigas M, Garcia-Martinez I, Pagerols M, Rovira P, Richarte V, et al. Epigenetic signature for attention-deficit/hyperactivity disorder: identification of miR-26b-5p, miR-185-5p, and miR-191-5p as potential biomarkers in peripheral blood mononuclear cells. Neuropsychopharmacology : official publication of the American College of Neuropsychopharmacology. 2018.
14. Garcia-Martinez I, Sanchez-Mora C, Pagerols M, Richarte V, Corrales M, Fadeuilhe C, et al. Preliminary evidence for association of genetic variants in pri-miR-34b/c and abnormal miR-34c expression with attention deficit and hyperactivity disorder. Transl Psychiatry. 2016;6(8):e879.
15. Zhu P, Pan J, Cai QQ, Zhang F, Peng M, Fan XL, et al. MicroRNA profile as potential molecular signature for attention deficit hyperactivity disorder in children. Biomarkers. 2022;27(3):230-9.
16. Gururajan A, Naughton ME, Scott KA, O'Connor RM, Moloney G, Clarke G, et al. MicroRNAs as biomarkers for major depression: a role for let-7b and let-7c. Transl Psychiatry. 2016;6(8):e862-e.
17. Mundalil Vasu M, Anitha A, Thanseem I, Suzuki K, Yamada K, Takahashi T, et al. Serum microRNA profiles in children with autism. Mol Autism. 2014;5(1):40.
18. Homorogan C, Enatescu VR, Nitusca D, Marcu A, Seclaman E, Marian C. Distribution of microRNAs associated with major depressive disorder among blood compartments. J Int Med Res. 2021;49(4):030006052110066.
19. Van Den Berg MMJ, Krauskopf J, Ramaekers JG, Kleinjans JCS, Prickaerts J, Briedé JJ. Circulating microRNAs as potential biomarkers for psychiatric and neurodegenerative disorders. Prog Neurobiol. 2020;185:101732.
20. Huang F, Long Z, Chen Z, Li J, Hu Z, Qiu R, et al. Investigation of Gene Regulatory Networks Associated with Autism Spectrum Disorder Based on MiRNA Expression in China. PLoS One. 2015;10(6):e0129052.
21. Hu Y, Ehli EA, Boomsma DI. MicroRNAs as biomarkers for psychiatric disorders with a focus on autism spectrum disorder: Current progress in genetic association studies, expression profiling, and translational research. Autism Res. 2017;10(7):1184-203.
22. Beveridge NJ, Cairns MJ. MicroRNA dysregulation in schizophrenia. Neurobiol Dis. 2012;46(2):263-71.
23. Camkurt MA, Karababa F, Erdal ME, Bayazıt H, Kandemir SB, Ay ME, et al. Investigation of Dysregulation of Several MicroRNAs in Peripheral Blood of Schizophrenia Patients. Clin Psychopharmacol Neurosci. 2016;14(3):256-60.
24. Geaghan M, Cairns MJ. MicroRNA and Posttranscriptional Dysregulation in Psychiatry. Biol Psychiatry. 2015;78(4):231-9.
25. Camkurt MA, Karababa İF, Erdal ME, Kandemir SB, Fries GR, Bayazıt H, et al. MicroRNA dysregulation in manic and euthymic patients with bipolar disorder. J Affect Disord. 2020;261:84-90.
26. Tonacci A, Bagnato G, Pandolfo G, Billeci L, Sansone F, Conte R, et al. MicroRNA Cross-Involvement in Autism Spectrum Disorders and Atopic Dermatitis: A Literature Review. Journal of Clinical Medicine. 2019;8(1):88.

27. Juvale IIA, Che Has AT. The Potential Role of miRNAs as Predictive Biomarkers in Neurodevelopmental Disorders. J Mol Neurosci. 2021.
28. Lee S-Y, Lu R-B, Wang L-J, Chang C-H, Lu T, Wang T-Y, et al. Serum miRNA as a possible biomarker in the diagnosis of bipolar II disorder. Sci Rep. 2020;10(1).
29. Mor M, Nardone S, Sams DS, Elliott E. Hypomethylation of miR-142 promoter and upregulation of microRNAs that target the oxytocin receptor gene in the autism prefrontal cortex. Mol Autism. 2015;6(1).
30. Levitskiy D, Confair A, Wagner KE, Devita S, Shea N, McKernan EP, et al. Longitudinal stability of salivary microRNA biomarkers in children and adolescents with autism spectrum disorder. Res Autism Spectr Disord. 2021;85:101788.
31. Beveridge NJ, Gardiner E, Carroll AP, Tooney PA, Cairns MJ. Schizophrenia is associated with an increase in cortical microRNA biogenesis. Mol Psychiatry. 2010;15(12):1176-89.
32. Ozkul Y, Taheri S, Bayram KK, Sener EF, Mehmetbeyoglu E, Öztop DB, et al. A heritable profile of six miRNAs in autistic patients and mouse models. Sci Rep. 2020;10(1).
33. Liu S, Zhang F, Wang X, Shugart YY, Zhao Y, Li X, et al. Diagnostic value of blood-derived microRNAs for schizophrenia: results of a meta-analysis and validation. Sci Rep. 2017;7(1).
34. Gou M, Pan S, Tong J, Zhou Y, Han J, Xie T, et al. Effects of microRNA-181b-5p on cognitive deficits in first-episode patients with schizophrenia: Mediated by BCL-2. J Psychiatr Res. 2021;136:358-65.
35. Moreau MP, Bruse SE, David-Rus R, Buyske S, Brzustowicz LM. Altered MicroRNA Expression Profiles in Postmortem Brain Samples from Individuals with Schizophrenia and Bipolar Disorder. Biol Psychiatry. 2011;69(2):188-93.
36. Marques FZ, Eikelis N, Bayles RG, Lambert EA, Straznicky NE, Hering D, et al. A polymorphism in the norepinephrine transporter gene is associated with affective and cardiovascular disease through a microRNA mechanism. Mol Psychiatry. 2017;22(1):134-41.
37. Horai T, Boku S, Okazaki S, Otsuka I, Ratta-Apha W, Mouri K, et al. miR-19b is elevated in peripheral blood of schizophrenic patients and attenuates proliferation of hippocampal neural progenitor cells. J Psychiatr Res. 2020;131:102-7.
38. Yoshino Y, Roy B, Dwivedi Y. Differential and unique patterns of synaptic miRNA expression in dorsolateral prefrontal cortex of depressed subjects. Neuropsychopharmacology. 2021;46(5):900-10.
39. Chen Y, Shi J, Liu H, Wang Q, Chen X, Tang H, et al. Plasma microRNA Array Analysis Identifies Overexpressed miR-19b-3p as a Biomarker of Bipolar Depression Distinguishing From Unipolar Depression. Front Psychiatry. 2020;11(757).
40. Ragusa M, Santagati M, Mirabella F, Lauretta G, Cirnigliaro M, Brex D, et al. Potential Associations Among Alteration of Salivary miRNAs, Saliva Microbiome Structure, and Cognitive Impairments in Autistic Children. Int J Mol Sci. 2020;21(17):6203.
41. Perkins DO, Jeffries CD, Jarskog LF, Thomson JM, Woods K, Newman MA, et al. microRNA expression in the prefrontal cortex of individuals with schizophrenia and schizoaffective disorder. Genome Biol. 2007;8(2):R27.
42. Shi W, Du J, Qi Y, Liang G, Wang T, Li S, et al. Aberrant expression of serum miRNAs in schizophrenia. J Psychiatr Res. 2012;46(2):198-204.
43. Ma J, Shang S, Wang J, Zhang T, Nie F, Song X, et al. Identification of miR-22-3p, miR-92a-3p, and miR-137 in peripheral blood as biomarker for schizophrenia. Psychiatry Res. 2018;265:70-6.
